# Synthesis, Thermal and Mechanical Properties of Nonisocyanate Thermoplastic Polyhydroxyurethane Nanocomposites with Cellulose Nanocrystals and Chitin Nanocrystals

**DOI:** 10.1021/acs.biomac.5c00113

**Published:** 2025-05-09

**Authors:** Pavithra M. Wijeratne, Connie Ocando, Bruno Grignard, Lars A. Berglund, Jean-Marie Raquez, Qi Zhou

**Affiliations:** † Division of Glycoscience, Department of Chemistry, School of Engineering Sciences in Chemistry, Biotechnology and Health, 7655 KTH Royal Institute of Technology, AlbaNova University Centre, 106 91, Stockholm, Sweden; ‡ Laboratory of Polymeric and Composite Materials, Department of Chemistry, Faculty of Science, 54521 University of Mons, Mons 7000, Belgium; § Center for Education and Research on Macromolecules (CERM), Federation of Researchers in Innovative Technologies for CO_2_ Transformation (FRITCO_2_T Research Platform), 26658 CESAM Research Unit, University of Liege, 13, Allée Du 6 août, Building B6a, Liege 4000, Belgium; ∥ Department of Fiber and Polymer Technology, KTH Royal Institute of Technology, Stockholm 100 44, Sweden

## Abstract

Incorporating biobased nanofillers including cellulose nanocrystals (CNCs) and chitin nanocrystals (ChNCs) into nonisocyanate polyurethane (NIPU) offers a multifunctional approach to improving mechanical and thermal properties while promoting sustainability and green chemistry. Nanocomposites of segmented thermoplastic polyhydroxyurethane (PHU) from vanillyl alcohol bis­(cyclocarbonate) (VABC), poly­(tetramethylene oxide) diamine (PTMODA), and bis­(aminomethyl) norbornane (NORB) reinforced with a low amount of CNCs and partially deacetylated ChNCs were prepared and characterized. Fourier transform infrared spectroscopy, atomic force microscopy, and small-angle X-ray scattering revealed that partially deacetylated ChNCs were covalently grafted to the PHU through aminolysis of carbonate end groups in the hard segment, while CNCs were mixed with the PHU without interfacial covalent bonding. Consequently, the PHU/ChNC nanocomposites showed nanophase separation with smaller hard domains compared to neat PHU, while the PHU/CNC nanocomposites exhibited a phase-mixed system with broader interface regions. Dynamic mechanical analysis and tensile tests further revealed that the PHU/ChNC nanocomposites demonstrated a 49-fold increase in Young’s modulus, a 20-fold increase in ultimate tensile strength, and a three-order-of-magnitude enhancement in storage modulus in the rubbery state compared to the PHU/CNC nanocomposites, highlighting the profound influence of interfacial covalent linkages in enhancing the thermal mechanical performance of segmented PHU.

## Graphical abstract

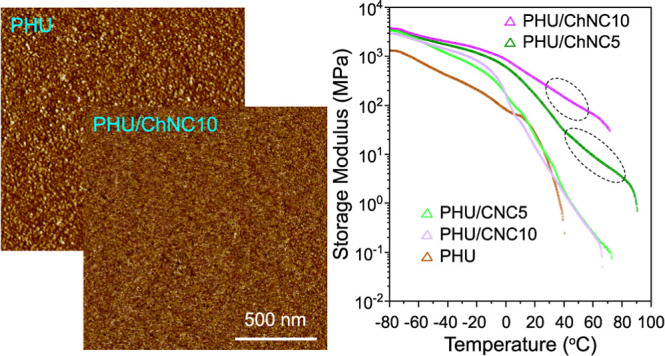

## 1 Introduction

The synthesis of poly­(hydroxyurethane)­s (PHUs) through aminolysis of cyclic carbonates (CC) has been proved to be a promising route to produce nonisocyanate polyurethanes (NIPU). This polyaddition reaction produces PHU analogous to conventional polyurethane (PU) but contains urethane bonds with additional primary and/or secondary hydroxy groups.^1^ Numerous studies have explored various approaches to PHU synthesis, including the preparation of cyclic carbonate-functionalized monomers,^2–6^ the exploitation of reaction catalysis,^7–9^ and the development of single-phase linear PHU^10–12^ and cross-linked PHU from renewable resources.^13–17^ However, investigations into segmented PHU structures, which closely resemble conventional thermoplastic polyurethane (TPU), remain relatively rare. The segmented structures are characterized by the coexistence of hard-segment (HS) and soft-segment (SS) domains, which exhibit phase separation due to their inherent incompatibility. The SS exists in a flexible, rubbery state, while the HS is either semicrystalline or glassy at room temperature. The stretchability of the segmented PHU is influenced by the SS, while the rigidity and strength are determined by the HS.^18^

Advancing structure–property relationship studies will facilitate the molecular design and synthesis of PHUs with improved properties, positioning them as viable alternatives to commercially available conventional PUs. Few studies have examined the synthesis, structure, and properties of segmented, nanophase-separated PHUs.^18–24^ Torkelson and co-workers reported the synthesis of segmented PHUs as potential thermoplastic elastomers using various polyether-based soft segments.^21,22,24^ Long and co-workers demonstrated the synthesis of poly­(hydroxyurethane-amide) from biobased precursors, producing segmented PHU with crystallizable hard domains.^25^ These investigations aimed to deepen the understanding of segmented PHU properties, particularly the influence of hydroxyl groups in PHU hard segments on the phase behavior and material properties, relative to conventional PUs. In addition, PHU-based nanocomposites reinforced with low fractions of nanoscale fillers offer several compelling advantages related to sustainability, mechanical performance, and functionality. Surface modifications of the nanofillers are required to increase the compatibility and their dispersion in the PHU matrix. Various surface-modified nanomaterials, including superhydrophobic silica nanoparticles,^26^ multiwalled carbon nanotubes functionalized with poly­(acryloyl carbonate),^27^ and gibbsite nanoplatelets functionalized with lysine,^28^ have been employed to enhance the thermal mechanical and flame-retardant properties of PHU.

Cellulose nanocrystals (CNCs), derived from controlled acid hydrolysis of cellulose fibers, exhibit appealing key properties including nanoscale dimensions, high surface area, unique morphology, low density, high specific strength and modulus, biodegradability, and an extremely low coefficient of thermal expansion.^29^ Similarly, chitin nanocrystals (ChNCs), extracted from native chitin found in the exoskeletons of arthropods such as crabs and shrimp, or pen, the internalized shell of squid, possess an equally impressive set of properties, including low toxicity, biocompatibility, biodegradability, and superior mechanical stiffness, among others.^30,31^ Previously, biobased nanofillers including CNCs, ChNCs, and nanoclays have been utilized to improve the mechanical, thermal, and barrier properties of conventional PUs.^32^ The hydrogen bonding in PHUs, originating from hydroxyl groups attached to urethane linkages, significantly enhances their material properties, including mechanical strength, elasticity, thermal stability, self-healing capability, and hydrophilicity.^33^ This inherent hydrophilicity facilitates the direct incorporation of hydrophilic fillers into the PHU matrix. These fillers are expected to synergistically enhance the nanocomposite’s properties, offering improved mechanical performance and tailored functionalities suitable for advanced applications.

In this study, we investigated several segmented PHU formulations by varying combinations of HS, SS, and chain extenders (CE) to evaluate their effects on the physical appearances, monomer conversions, and molar masses. Based on these criteria, the optimal PHU formulation was selected for nanocomposite preparation. Nanoscale rod-like CNCs and partially deacetylated ChNCs were incorporated into the segmented PHU formulation to enhance the thermal and mechanical properties at low nanocrystal loadings. The partial deacetylation of ChNCs introduced a higher amount of amine groups on their surface than the extracted chitin from squid, facilitating covalent linkage formation with the cyclic carbonate end groups of PHU. This enhanced compatibility between ChNCs and the PHU matrix was expected to yield a much stronger reinforcing effect in the PHU/ChNC nanocomposites. The structure, nanophase separation, thermal degradation, and mechanical and thermal mechanical properties of the PHU/CNC and PHU/ChNC nanocomposites were investigated and compared.

## 2 Experimental Section

### 2.1 Materials

Vanillyl alcohol bis­(cyclocarbonate) (VABC) with a carbonate content of 5.8 mequiv/g was purchased from Specific Polymers. Bisphenol A diglycidyl ether-derived dicyclocarbonate (BPADC) with a carbonate content of 4.4 mequiv/g was synthesized in-house through the coupling of CO_2_ with epoxides. Polypropylene glycol diamine (PPGDA, average *M*_n_ of 2000 g/mol, Sigma-Aldrich 406,686), *O*,*O*′-bis­(2-aminopropyl) polypropylene glycol-*block*-polyethylene glycol-*block*-polypropylene glycol (Jeffamine ED-600 and Jeffamine ED-900, Sigma-Aldrich 14,526 and 14,527), *m*-xylylenediamine (*m*-XDA, Sigma-Aldrich X1202), 1,5-diamino-2-methylpentane (Dytek-A, Sigma-Aldrich 329,665), 1,8-diazabicyclo[5.4.0]­undec-7-ene (DBU, Sigma-Aldrich 139,009), anhydrous dimethylformamide (DMF), sodium chloride (NaCl), sodium chlorite (NaClO_2_), hydrochloric acid (HCl), sodium hydroxide (NaOH), and deuterated chloroform (CDCl_3_) were purchased from Merk KGaA, Germany. Bis­(aminomethyl) norbornane (NORB, a mixture of isomers, TCI B3852) was purchased from TCI America. Poly­(tetramethylene oxide) diamine (PTMODA, Elastamine HT-1700) and polyethylene glycol diamine (PEGDA, Elastamine HE-1700), both with an average *M*_n_ of 1700 g/mol, were kindly provided by Huntsman Corporation. Cellulose nanocrystals (CNCs, 0.85 wt % sulfur on dry CNC sodium) from wood cellulose were purchased from the University of Maine, USA. All chemicals and materials were used as received.

### 2.2 Preparation of Chitin Nanocrystals (ChNCs)

Partially deacetylated ChNCs were prepared from squid pen through a multistep process.^34,35^ In brief, the raw powdered squid pen sample was demineralized in 1 M HCl at room temperature for 12 h, followed by thorough rinsing with deionized water. Subsequently, the sample was treated in 1 M NaOH at room temperature for 12 h to remove the protein and rinsed again with deionized water. Such acid–base treatments were repeated three times, and the isolated β-chitin was dried at 60 °C in a vacuum oven before further partial deacetylation. To increase the content of amine groups, the isolated β-chitin (1 g) was suspended in 33 wt % NaOH (25 mL) and heated at 90 °C for 4 h with stirring. The partially deacetylated chitin was collected and thoroughly washed with deionized water through repeated centrifugation at 4100*g* for 15 min until neutral pH was reached. The deacetylated chitin was suspended in water and mechanically disintegrated through repeated ultrasonication (Branson SFX550 Sonifier) until a turbid supernatant was formed, representing a stable aqueous suspension of deacetylated ChNCs. The CNC and ChNC samples were freeze-dried from a 0.05 wt % aqueous suspension and redispersed in DMF by probe sonication at 25% output for 2 min (Branson SFX550 Sonifier) without any additives or chemical modification.

### 2.3 Synthesis of Segmented PHUs

The segmented PHUs were synthesized by a reaction between the bis­(cyclic carbonates) (either VABC or BPADC) and various long-chain and short-chain diamines, representing the hard segment (HS), soft segment (SS), and chain extender (CE), as illustrated in Figure 1a. The HS content in the segmented PHU was calculated from the total weight of the bis-carbonate and the chain extender and was maintained at 50 wt % for all 15 PHU formulations, as shown in Table 1. In a typical synthesis of segmented PHU from VABC, PTMODA, and NORB, the bis-carbonates VABC (1.3209 g, 3.73 mmol) and the long-chain diamine Elastamine HT-1700 (1.7604 g, 1.035 mmol) were dissolved in 1.68 mL of anhydrous DMF with the addition of the catalyst DBU (0.0556 mL, 0.37 mmol) in a 20 mL scintillation vial. The mixture had a carbonate concentration of 0.70 M. After the mixture was kept at 80 °C with stirring for 8 h, the chain extender NORB (0.4153 g, 2.69 mmol) was added, and the reaction was further kept for 16 h. Subsequently, the mixture was degassed for 30 min under vacuum and then cast and cured on a Teflon mold at 80 °C in a vacuum oven for 48 h to remove the solvent DMF. Finally, the sample was hot-pressed at 100 °C for 5 min to produce a film with a thickness of ca. 1 mm. The obtained PHU samples were characterized by using proton nuclear magnetic resonance (^1^H NMR) spectroscopy and size exclusion chromatography (SEC) to determine the monomer conversion and polymer molecular weights, respectively, in order to select a formulation for the preparation of the nanocomposite with CNCs and ChNCs.

**Figure 1:**
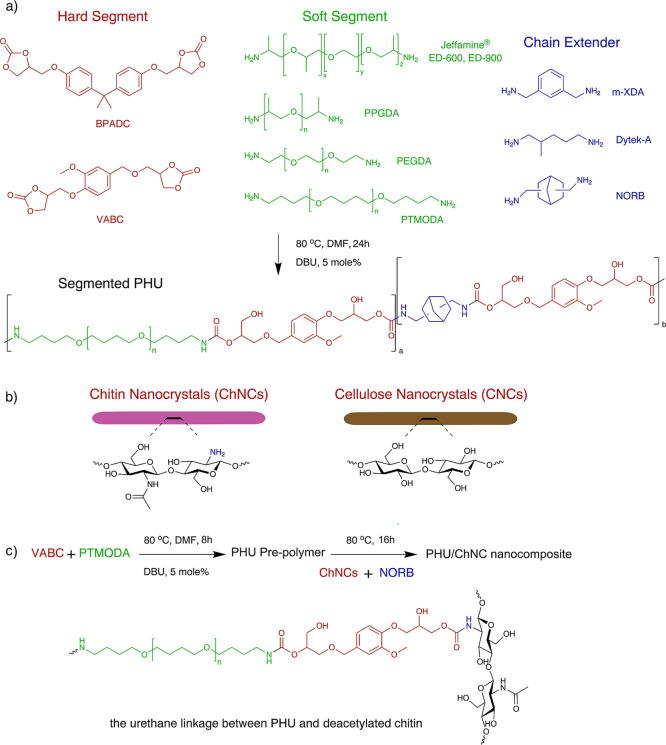
(a) Reaction scheme for the synthesis of segmented PHUs with VABC and BPADC as the hard segment and different diamines as the soft segment and chain extender and the possible structure of segmented PHU from VABC, PTMODA, and NORB. (b) The chemical structure of cellulose nanocrystals (CNCs) and partially deacetylated chitin nanocrystals (ChNCs). (c) Reaction scheme for the synthesis of PHU/ChNC nanocomposites and the representative urethane linkage between PHU and deacetylated chitin.

**Table 1:** Summary of the Properties of PHU Samples

samples	molar ratio	conversion (%)^a^	*M*_w_ (g/mol)^b^	*M*_n_ (g/mol)^b^	PDI^c^
VABC/ED-600/m-XDA	1.5/1/0.5	93	22,000	12,000	1.8
VABC/ED-900/m-XDA	2/1/1	93	32,000	15,500	2.1
VABC/PPGDA/m-XDA	0.9/0.2/0.7	92	18,000	11,000	1.7
VABC/ED-600/NORB	1.5/1/0.5	93	24,000	13,000	1.9
VABC/ED-900/NORB	2/1/1	93	30,000	15,000	2.0
VABC/PPGDA/NORB	4.5/1/3.5	92	19,000	10,000	1.9
VABC/ED-600/Dytek-A	1.5/1/0.5	93	30,000	13,000	2.3
VABC/ED-900/Dytek-A	2/1/1	93	34,000	15,000	2.3
VABC/PPGDA/Dytek-A	4.5/1/3.5	93	22,000	11,500	1.9
VABC/PTMODA/Dytek-A	3.8/1/2.8	93	40,000	12,000	3.3
VABC/PEGDA/Dytek-A	3.8/1/2.8	93	33,000	14,000	2.3
VABC/PTMODA/NORB	3.6/1/2.6	93	70,000	16,000	4.3
VABC/PEGDA/NORB	3.6/1/2.6	92	50,000	15,000	3.3
BPADC/PTMODA/NORB	3.6/1/2.6	87	67,000	13,000	5.0
BPADC/PEGDA/NORB	3.6/1/2.6	86	33,000	13,000	2.6

a Monomer conversion of bis­(cyclic carbonate) determined by ^1^H NMR.

b *M*_n_ and *M*_w_ determined by SEC.

c Polydispersity index, PDI = *M*_w_/*M*_n_.

### 2.4 Preparation of PHU/CNC and PHU/ChNC Nanocomposites

The preparation procedure for the nanocomposite films followed the synthesis steps of the chosen segmented PHU (VABC/PTMODA/NORB) as shown in Figure 1b. The dispersions of CNCs or ChNCs in DMF were added with the chain extender NORB to the VABC/PTMODA prepolymer. The resulting polymer mixture had a solid content of 49% in DMF. The nanocomposite films with different compositions were successfully prepared by altering the CNC or ChNC content as 2, 5, and 10 wt % of the total material. The obtained nanocomposite films were coded as PHU/CNC2, PHU/CNC5, PHU/CNC10, PHU/ChNC2, PHU/ChNC5, and PHU/ChNC10.

### 2.5 Characterizations

The morphology of the discrete CNCs and ChNCs, as well as the cross-sectional surfaces of both neat PHU and the nanocomposite films, was visualized using atomic force microscopy (AFM). ScanAsyst mode was used on a Nanoscope IIIa, Multimode, Digital Instruments, with an integrated force generated by cantilever/silicon probes with a tip radius of 2–12 nm and a resonance frequency of 70 kHz. Prior to imaging, 0.01 wt % aqueous colloidal suspensions of CNCs and ChNCs were drop-cast onto clean mica surfaces and allowed to dry at ambient conditions. The neat PHU and the nanocomposite samples were embedded in an acrylic resin bisphenol A ethoxylate diacrylate ABPE-10 (Shin-Nakamura Chemical Co. Ltd., Japan), cured with UV for 1 min, and cut with a microtome to obtain the cross-sectional surfaces. X-ray diffraction (XRD) patterns of the CNC and ChNC samples were recorded by an X’Pert Pro diffractometer (model PW 3040/60) in the reflection mode (5–50° 2θ angular range, steps 0.05°). Freeze-dried samples were pressed into pellets for measurements with rotation using a position-sensitive detector. The CuKr radiation source (λ = 1.5418 Å) was operated at 45 kV and 40 mA and monochromatized using a 20 μm Ni filter. The crystallinity index (CrI) was calculated based on the intensities of crystalline and amorphous peaks using the following equation:^36,37^

$$CrI=\frac{I_{2\theta }-I_{am}}{I_{2\theta }}\times 100\%$$

where *I*_2θ_ refers to the overall intensity of the crystalline lattice diffraction peaks, observed at 19.2° for ChNCs and 22.4° for CNCs. *I*_am_ denotes the intensity of the amorphous diffractions measured at approximately 16.0° for ChNCs and 18.2° for CNCs.

In order to determine the amine content of the partially deacetylated ChNCs, a dried sample (0.1 g) was added to water (60 mL) with pH adjusted to 9 using 0.5 M NaOH. The mixture was stirred for 30 min to prepare a well-dispersed slurry, and then 0.1 M HCl was added to adjust the pH to 2.5–3.0. The suspension was titrated with 0.05 M NaOH solution at a speed of 0.1 mL/min until the pH value reached 11, and the conductivity was monitored by using a conductometric station (SevenCompact, Mettler–Toledo). High-resolution magic angle spinning (HR MAS) ^1^H NMR was conducted to verify the results of titration. The sample was prepared in a disposable rotor insert (Bruker B4493 Kel-F inserts for 4 mm MAS rotor) by adding 10 mg of freeze-dried ChNCs swollen in 25 μL of 20 wt % DCl solution in D_2_O. HR MAS ^1^H NMR spectra were recorded at ambient temperature on a Bruker Avance III 500 spectrometer equipped with a ^1^H/^13^C HR MAS probe. The sample was spun at 5 kHz around its own axis, and 1D spectra were recorded with 64 scans.

The monomer conversion in the synthesis of the segmented PHUs was determined by analyzing the peak integrals of ^1^H NMR spectra that were obtained using a Bruker Avance III 400 MHz NMR spectrometer with a direct cryoprobe at room temperature and deuterated chloroform (CDCl_3_) as a solvent. The number average molecular weight (*M*_n_) and weight average molecular weight (*M*_w_) of the PHUs were determined by SEC, which was performed in DMF at 50 °C using an Agilent liquid chromatography system equipped with an Agilent degasser, an isocratic HPLC pump (flow rate = 0.7 mL/min), an Agilent autosampler (loop volume = 100 μL, solution conc. = 1 mg/mL), an Agilent-DRI refractive index detector, and three columns: a PL gel 10 μm guard column and two PL gel Mixed-D 10 μm columns (linear columns for separation of MWPS ranging from 500 to 10^7^ g/mol). Molecular weight calibration was performed by using polystyrene standards.

Fourier transform infrared spectroscopy (FTIR) was conducted using a PerkinElmer Spectrum 2000 FTIR instrument equipped with an MKII Golden Gate single reflection attenuated total reflectance (ATR) system (Specac Ltd., London, UK). The ATR crystal used was an MKII heated diamond 45 ATR top plate. Cross-sectional morphologies of the neat segmented PHU, PHU/CNC10, and PHU/ChNC10 nanocomposite films were examined by using field-emission scanning electron microscopy (FE-SEM). Imaging was conducted with a Hitachi S-4800 operated at accelerating voltages of 1 and 3 kV, a probe current of 10 μA, and a working distance of approximately 8–9 mm. Fracture surfaces were prepared by freeze-fracturing the samples in liquid nitrogen, followed by mounting them in a thin specimen split mount holder. Prior to imaging, a 5 nm thick gold–platinum (Au/Pt) conductive layer was deposited onto the sample surfaces using a JFC1300 sputter coater. Small-angle X-ray scattering (SAXS) measurements were performed using a SAXSpoint 2.0 system (Anton Paar, Austria) equipped with an Eiger R 1 M Horizontal detector (Dectris, Switzerland) and a microfocus X-ray source (Cu Kα radiation, wavelength 1.5418 Å). The sample-to-detector distance was set to 575.7 mm, and all measurements were performed at ambient temperature. Thermogravimetric analysis (TGA) was carried out using a Mettler Toledo TGA/DSC STARe system with nitrogen as the purge gas at a flow rate of 50 mL/min. Approximately 10 mg samples were subjected to heating from 25 to 650 °C at a rate of 10 °C/min. The mechanical properties of the nanocomposite films were evaluated by using a universal material testing machine (Instron 5944, UK), equipped with a 500 N load cell. Rectangular strips with dimensions of 30 × 5 × 1 mm^3^ were cut from the samples in a single stroke using a microtome blade (Leica High-profile disposable blades DB80HS) and a hammer. The specimens were conditioned at a relative humidity (RH) of 50% at 22 °C for 2 days prior to testing. The tensile test was conducted at ambient temperature using a gauge length of 20 mm and a crosshead speed of 100 mm/min. Five strips were tested for each sample. Young’s modulus was determined by calculating the slope in the initial low strain region. Dynamic mechanical analysis (DMA) was carried out using a TA Instruments Q800 in tension mode, with a testing frequency of 1 Hz and a heating rate of 3 °C/min from −80 to 100 °C.

## 3 Results and Discussion

### 3.1 Characterization of the Segmented PHUs

The reaction kinetics, conversions, and molar masses are critical parameters that directly influence the properties of NIPUs.^38,39^ In order to find an optimal segmented PHU matrix for nanocomposite preparation with CNCs and ChNCs, various samples were synthesized from two bis­(cyclic carbonates) VABC and BPADC, five long-chain diamines Jeffamine ED-600 (*M*_n_ of 600 g/mol), Jeffamine ED-900 (*M*_n_ of 900 g/mol), PPGDA (*M*_n_ of 2000 g/mol), PEGDA (*M*_n_ of 1700 g/mol), and PTMODA (*M*_n_ of 1700 g/mol), and three short-chain diamine extenders *m*-XDA, Dytek-A, and NORB. To effectively isolate and interpret the filler-induced effects, it was critical to maintain the polymer matrix in a thermoplastic elastomeric state, which inherently requires discrete hard segments dispersed within a continuous rubbery soft segment matrix. In the previous studies, Nanclares et al.^18^ demonstrated that among various PHU formulations tested, only the one with 50 wt % hard segments exhibited elastomeric behavior, characterized by an elongation at break of approximately 650%. In contrast, increasing the hard-segment content to 70 wt % resulted in a glassy, brittle polymer with an elongation at break of only 6%, attributed to the development of a continuous hard-phase morphology with dispersed soft segments. Similarly, Beniah et al.^22^ found that elastomeric tensile responses were observed in PHU formulations with hard-segment contents of 50 wt % or less. When the hard-segment content was increased to 60 wt %, a pronounced yield point and changes in the nanostructure were observed, indicating the formation of interconnected, continuous hard domains rather than discrete segments embedded in a soft matrix. Therefore, in this study, the molar ratio between bis­(cyclic carbonate), long-chain diamine, and chain extender was adjusted accordingly to make the hard segment content 50% in all PHU samples (Table 1). All samples presented a slightly yellowish and translucent appearance. The samples based on PPGDA, PEGDA, and PTMODA with relatively higher *M*_n_ exhibited solid states, while the samples based on Jeffamine showed highly viscous liquid characteristics (Figure S1). The monomer conversion of bis­(cyclic carbonates) was determined by ^1^H NMR (Figures S2–S16), and the number average molecular weight (*M*_*n*_) and weight average molecular weight (*M*_w_) obtained from SEC (Figure S17) are summarized in Table 1. The monomer conversion was consistent at 92–93% for VABC-based PHUs, while BPADC-based PHUs had a lower conversion of 86–87%. In terms of molecular weight, VABC-based PHU samples showed relatively higher *M*_*n*_ than those for BPADC-based PHUs, as well as those reported in literature for segmented PHUs, which were derived from 1,3/1,4-divinylbenzene dicyclocarbonate, terephthalic biscyclocarbonate, and resorcinol biscyclocarbonate.^19,20,23,24^ The soft segment long-chain diamine also played a crucial role in the molecular weight of segmented PHU. Specifically, PTMO-based PHU samples demonstrated significantly elevated *M*_n_, however, with a slightly higher dispersity (PDI). Based on the highest conversion (93%) and the highest *M*_w_ (70,000 g/mol), the formulation with VABC as the hard segment, PTMO as the soft segment, and NORB as the chain extender was selected as the matrix for further nanocomposite preparation with CNCs and ChNCs.

### 3.2 Partial Deacetylation of ChNCs

The successful partial deacetylation of the squid ChNCs was confirmed by using ATR-FTIR spectroscopy (Figure S18). The spectra exhibited a decrease and split of the peak at 1624 cm^–1^, corresponding to amide carbonyl stretching. Typically, a larger peak area at this wavenumber indicates a higher content of acetylated glucosamine in chitin. Therefore, the observed reduction in the peak area confirmed the successful deacetylation of *N*-acetyl-d-glucosamine in chitin. The amino group content of the deacetylated ChNCs was determined using conductometric titration to be 1.85 mmol/g, corresponding to a degree of deacetylation (DDA) of 35%. The native squid chitin had an amino group content of 0.8 mmol/g and a DDA value of 16%. According to the method reported by Dahmane et al.,^40^ the DDA value was further confirmed by analyzing the peak integrals corresponding to the anomeric proton H-1 of the acetylated and deacetylated units from the HR MAS ^1^H NMR spectra of the partially deacetylated squid ChNCs using concentrated DCl as a solvent (Figure S19). The obtained DDA value was 34%, higher than that (26–30%) of partially deacetylated α-chitin from crab shell.^35^

The dimensions and shape of the partially deacetylated squid ChNCs were characterized using AFM as compared to those of the wood CNCs (Figure S20). The AFM height images revealed that both types of nanofillers exhibited typical needle-like structure. Both CNCs and ChNCs displayed a tendency to form laterally aggregated particles and clusters, a phenomenon occurring during the drying step on the mica surface, consistent with those reported in literature.^41,42^ The CNCs showed a length ranging from 70 to 330 nm and a width from 4 to 14 nm, while the length of ChNCs ranged from 100 to 500 nm and their width from 3 to 13 nm. As calculated from the peak length and width, the aspect ratio (length-to-width ratio) of the ChNCs was 31, higher than that of the CNCs, which was 19. The crystal structures of the CNCs and ChNCs were characterized with XRD analysis (Figure S21). The CNCs exhibited characteristic diffraction peaks at 2θ of 14.7°, 16.4°, 22.6°, and 34.5°, which are assigned to the crystalline planes for cellulose I with Miller indices of (11̅0), (110), (200), and (004), respectively.^43,44^ The diffractogram of the ChNCs displayed two broad peaks at 2θ of 8.22° and 19.32°, corresponding to the crystal planes (010) and (110) of β-chitin, respectively.^34^ The CrI was 82% for CNCs and 69% for the ChNCs. The native squid chitin exhibited a CrI of 81%, which indicates that partial deacetylation resulted in reduced crystallinity in chitin.

### 3.3 PHU/CNC and PHU/ChNC Nanocomposites

The PHU/CNC and PHU/ChNC nanocomposites have been fabricated by a two-step methodology consisting of the synthesis of cyclic carbonate telechelic PHU prepolymers using long-chain diamines followed by their chain extension by reaction with blends of CNC or ChNC with short-chain diamines. All samples presented a slightly yellowish and translucent nature, gradually increasing with higher filler content (Figure S22). The polymerization process was slightly affected by the presence of nanofillers, as evidenced by ^1^H NMR analysis, which revealed a cyclic carbonate monomer conversion of 87% for both PHU/CNC10 and PHU/ChNC10 (Figure S23). However, it is important to note that complete dissolution of the nanocomposite samples in CDCl_3_ was not achieved, likely due to the presence of CNCs and covalently grafted ChNCs within the polymer matrix. Figure 2a shows the FTIR spectra of the starting monomers VABC and PTMODA, the prepolymer, the chain extender NORB, and the resulting segmented PHU. The successful formation of cyclic carbonate-terminated prepolymer was evident by the presence of a urethane carbonyl peak at 1696 cm^–1^ and excess carbonate at 1781 cm^–1^. The strong peak at 1110 cm^–1^ was assigned to the C–O ether stretching, indicating the effective incorporation of the long-chain diamine PTMODA into the prepolymer. Following the addition of chain extender NORB, the segmented PHU exhibited an almost complete disappearance of the carbonate peak of VABC at 1781 cm^–1^, indicating the conversion of carbonate functional groups into urethane linkages by the short-chain diamine-based chain extender. The presence of urethane carbonyl stretches at 1696 cm^–1^ and the hydroxy and N–H groups at 3200–3600 cm^–1^ further confirmed the formation of hydroxyurethane. The broad peak associated with hydrogen-bonded urethane carbonyls at 1696 cm^–1^ was also evident as peaks in the range of 1710–1730 cm^–1^ for non-hydrogen-bonded urethane carbonyl groups were not detected.^20,21,24^ In addition, the double peak at 2850 cm^–1^ and 2915 cm^–1^ was attributed to the symmetric and asymmetric stretching of C–H bonds in the PHU, and the peak at 1534 cm^–1^ was attributed to the N–H bending of urethane linkages. Notably, the formation of urea carbonyl was not detected in the carbonyl regions, which typically appear at 1640 cm^–1^ and 1670 cm^–1^ for hydrogen-bonded and non-hydrogen-bonded urea, respectively.^45^

**Figure 2:**
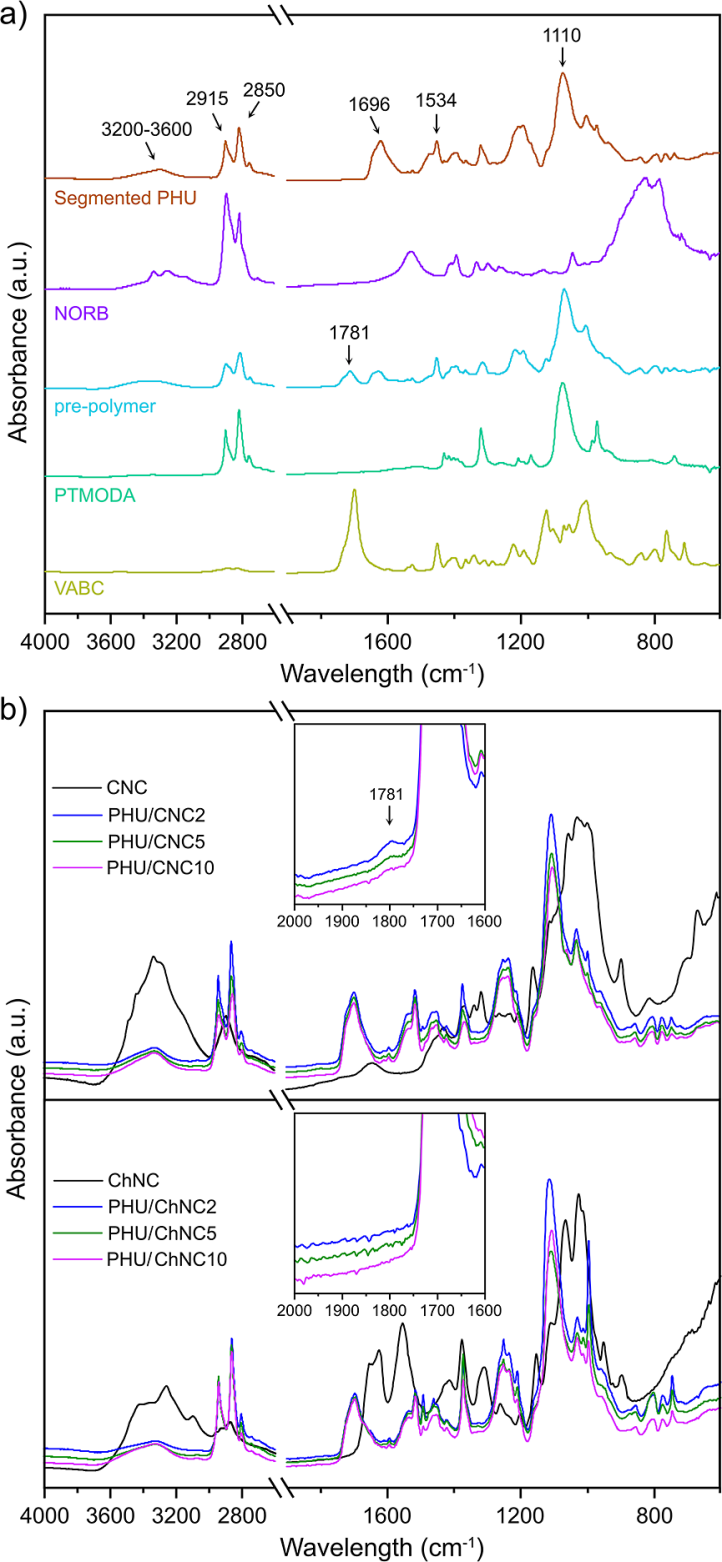
(a) Stacked ATR–FTIR spectra of VABC, PTMODA, NORB, the PHU prepolymer, and the segmented PHU; (b) overlaying ATR–FTIR spectra of the CNC and PHU/CNC nanocomposites and the ChNC and PHU/ChNC nanocomposites.

To prepare the PHU/CNC and PHU/ChNC nanocomposites, different amounts of nanofillers were added with the chain extender NORB to the VABC/PTMODA prepolymer. All the PHU/CNC and PHU/ChNC nanocomposites exhibited an almost complete disappearance of the band at 1781 cm^–1^, attributed to the carbonyl (C=O) group of the VABC monomer, while the urethane carbonyl (C=O) band at 1696 cm^–1^ signified successful polymerization (Figure 2b). Particularly, the absence of peaks within the range of 1710–1730 cm^–1^, which are attributed to non-hydrogen-bonded carbonyl groups,^19,20,24^ provided compelling evidence that almost all carbonyl groups participate in hydrogen bonding interactions in the nanocomposites. Such interactions occurred not only within the PHU matrix but also with the abundant hydroxyl groups present on the surfaces of CNC and ChNC. The broad peak observed in the range 3200–3600 cm^–1^ corresponds to N–H and hydroxy groups in the PHU and ChNC, as well as the hydroxy groups in the nanofillers CNC and ChNC. A zoomed-in FTIR plot (Figure 2b) revealed a subtle peak at 1781 cm^–1^, corresponding to the carbonate carbonyl end groups in the PHU/CNC nanocomposites. In contrast, the ChNC-based nanocomposites exhibited no peak in this region (Figure 2b), confirming a reaction between the cyclic carbonate end groups in the polymer matrix and amine groups on partially deacetylated ChNC upon curing (Figure 1c). As a result, the PHU polymer chains were grafted onto the ChNC nanoparticles, transforming them from passive reinforcements into integral components of the overall nanocomposite structure with increased interfacial compatibility. On the other hand, apart from hydrogen bonding, no evident covalent bonding was found between CNC and the PHU polymer matrix, similar to the composite of PHU with natural cellulose fibers.^46^

The microstructure and nanophase separation between the soft and hard segments in the neat PHU, PHU/CNC, and PHU/ChNC nanocomposites were investigated using FE–SEM (Figure 3) and AFM (Figure 4a). As shown in Figure 3, the ChNC nanoparticles in the PHU/ChNC10 nanocomposite were well-dispersed and uniformly distributed throughout the polymer matrix. Overall, the CNC nanoparticles were also well-dispersed in the PHU/CNC10 nanocomposite. However, some small aggregates composed of clustered CNCs were observed. The superior dispersion of ChNCs is attributed to effective covalent grafting with the PHU matrix, facilitated by reactive sites on partially deacetylated ChNCs. This chemical interaction promotes the stable and homogeneous dispersion of ChNCs. In contrast, CNCs primarily interact with the PHU matrix through weaker hydrogen bonding, which is less effective in preventing aggregation, resulting in the formation of small CNC clusters. The inphase AFM images of the cross-sectional surfaces generated by microtome cutting revealed two distinct phases of different contrasts in both neat PHU and the nanocomposites. This phase separation is attributed to the inherent thermodynamic incompatibility between the soft and hard segments, arising from differences in terms of mechanical and viscoelastic properties for both segments.^47,48^ The continuous dark areas indicate soft domains formed by long-chain flexible diamines with a lower modulus. In contrast, the nanoscale bright regions represent self-assembled hard domains formed by the urethane segment of aromatic bis­(cyclocarbonate)­s VABC and short-chain diamine NORB through hydrogen bonds. The coexistence of these soft, hard, and transition domains clearly demonstrated nanophase separation. Domain size distribution histograms derived from the AFM data are provided in Figure S24. The neat segmented PHU showed homogeneously distributed hard domains (bright, spherical-shaped areas) with an average diameter of 9.9 ± 1.8 nm. The PHU/ChNC nanocomposites exhibited homogeneously distributed hard domains of reduced sizes, with average diameters of 8.9 ± 1.2, 8.4 ± 1.4, and 8.2 ± 1.0 nm for nanocomposites with 2, 5, and 10 wt % ChNC, respectively. This decrease is attributed to the covalent linking of ChNC nanoparticles to the hard segments of the segmented PHU, restricting their assembly into larger domains (Figure 4b). The PHU/CNC nanocomposites showed hard domain sizes of 11.4 ± 2.3 and 11.5 ± 1.9 nm with the addition of 2 and 5 wt % CNC while a slight decrease to 9.4 ± 1.4 nm with 10 wt % CNC. However, these hard domains were rather heterogeneous in size and distribution as compared to the neat PHU and the PHU/ChNC nanocomposites. This effect is attributed to the fact that CNCs formed hydrogen bonds with both hard and soft segments of PHU, leading to greater mixing between the interfaces. The interfaces between hard and soft domains became less discernible, aligning with those CNC/polyurethane nanocomposites reported in literature.^49–51^

**Figure 3:**
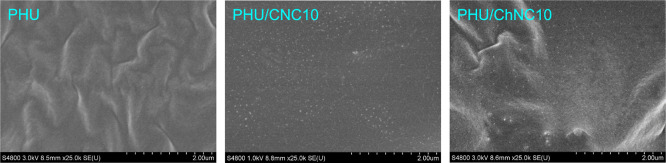
FE–SEM images of the freeze-fractured cross-sectional surfaces of the neat segmented PHU, PHU/CNC10, and PHU/ChNC10 nanocomposite films.

**Figure 4:**
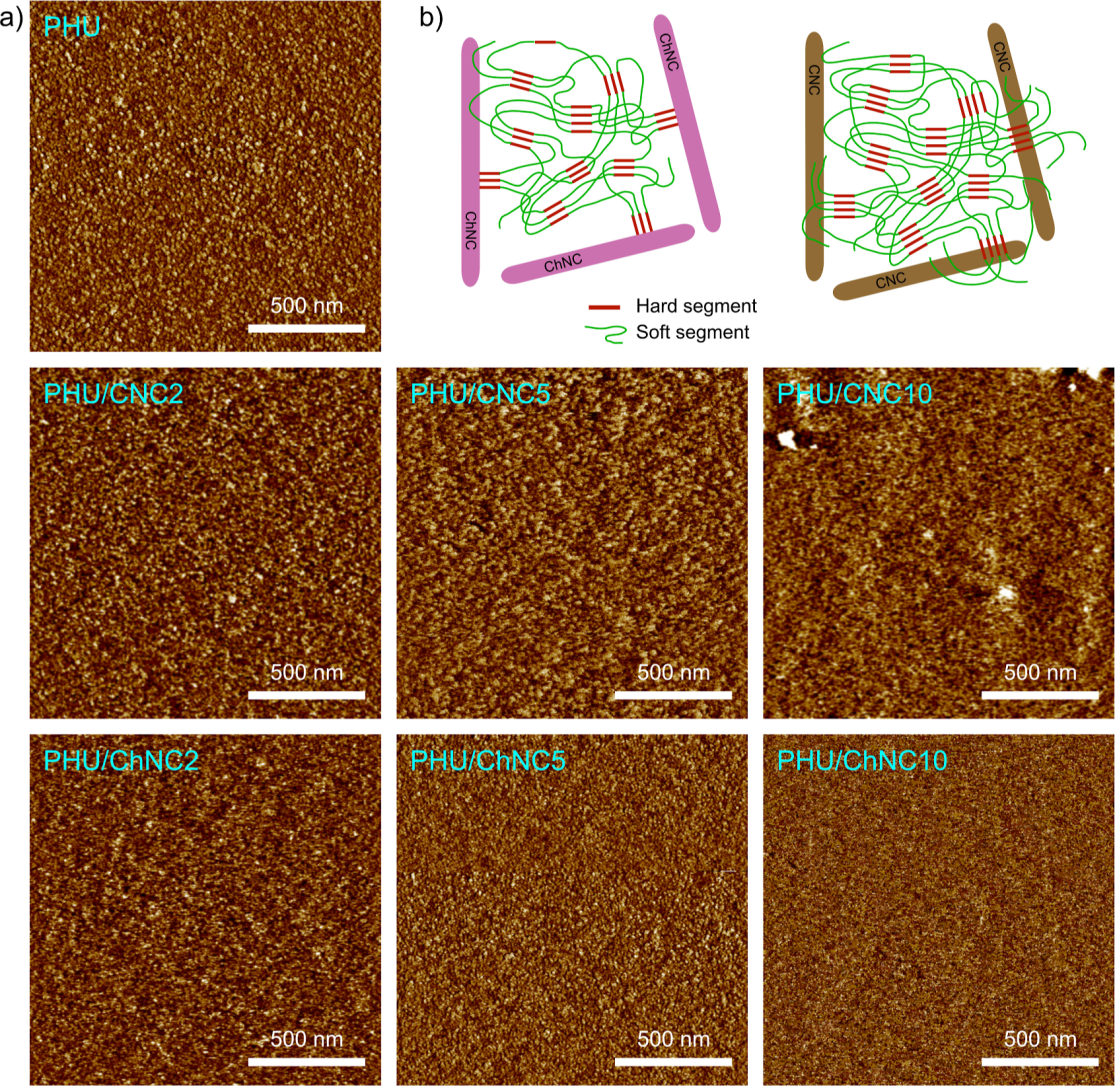
(a) AFM inphase images of the cross-sectional surfaces of the neat segmented PHU and the PHU/CNC and PHU/ChNC nanocomposites generated by microtome cutting after embedding in acrylic resin. (b) Schematic cartoon illustrating the integration of ChNCs and CNCs in the nanophase-separated PHU.

SAXS was used to further understand the impact of nanofillers CNC and ChNC on nanophase separation in segmented PHU. SAXS patterns of the neat PHU and the PHU/CNC and PHU/ChNC nanocomposites are shown in Figure 5. The neat PHU showed a typical single broad interference peak, indicating a significant nanophase separation with an interdomain spacing (*d*-spacing) of 11.8 nm. Similar single interference peaks were observed in the PHU/ChNC nanocomposites, which shifted to larger scattering vector (*q*) values with increasing ChNC content. The corresponding *d*-spacings were 11.4, 10.8, and 10.1 nm for PHU/ChNC2, PHU/ChNC5, and PHU/ChNC10, respectively. In contrast, the PHU/CNC nanocomposites exhibited rather broader and less pronounced scattering peaks that shifted to smaller *q* values, indicating less order in phase-separated domains and increased spacing between them. The corresponding *d*-spacings were 18.1, 16.7, and 10.9 nm for PHU/CNC2, PHU/CNC5, and PHU/CNC10, respectively. This result implies that CNCs are well dispersed throughout the PHU matrix, forming hydrogen bonds with both hard and soft domains, leading to a phase-mixed system with broader interface regions, which is consistent with the AFM observations.

**Figure 5:**
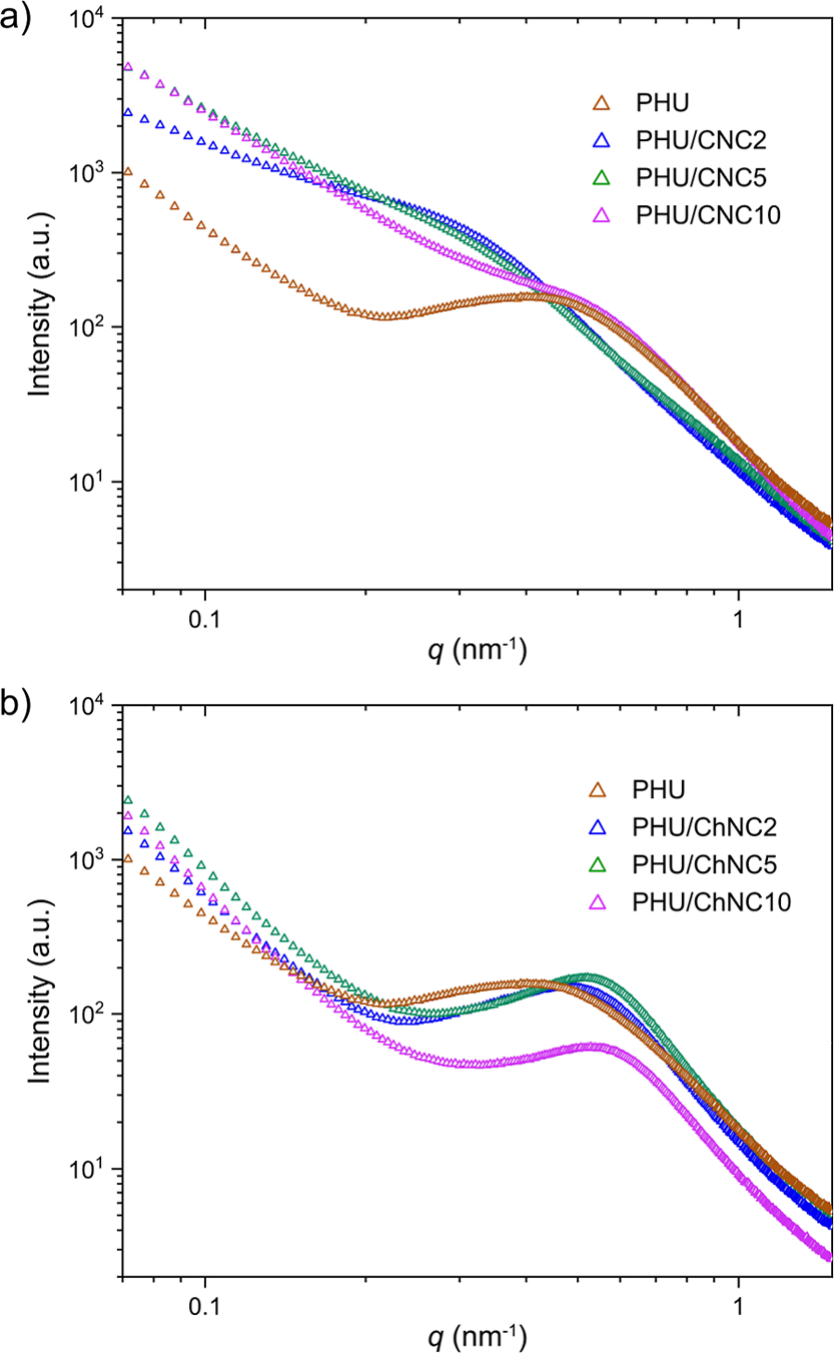
Small-angle X-ray scattering (SAXS) patterns of (a) PHU/CNC and (b) PHU/ChNC nanocomposites as compared to the neat PHU.

### 3.4 Thermal and Mechanical Properties

Figure 6 presents the TGA and DTG curves, illustrating the thermal stability and decomposition behavior of PHU/CNC and PHU/ChNC nanocomposites. The temperatures at 5% mass loss (T_5%_), the maximum mass loss temperatures (*T*_max1_ and *T*_max2_), and the char residues at 600 °C for the neat PHU and nanocomposite samples are summarized in Table S1. The neat segmented PHU exhibited two degradation stages located at 324 °C (*T*_max1_) and 417 °C (*T*_max2_), corresponding to the degradations of hard and soft segments, respectively, as thermal stability of the urethane groups is relatively lower.^52,53^ The weight losses for the first and second stages were 49.7 and 47.7 wt %, respectively, with a char residue of 2.5% at 600 °C, consistent with the formulation of the segmented PHU. The introduction of CNC and ChNC did not change the two-step degradation process. Interestingly, the degradation peak of the hard segment was broadened, and the *T*_max1_ value increased up to 350 °C with CNC and ChNC contents in both nanocomposites, while the degradation of the soft segment and *T*_max2_ values were not affected when compared with that of the neat PHU (Table S1). This indicates the strong interaction between the nanofillers and the hard segment of the PHU. Although the neat CNCs exhibited lower thermal stability compared to the neat ChNCs, the PHU/CNC10 nanocomposites demonstrated a T_5%_ value of 297 °C. This value surpassed that of neat PHU (290 °C) and PHU/ChNC10 (283 °C), highlighting the importance of interfacial hydrogen bonding between the nanofillers and the PHU matrix in enhancing thermal stability. The PHU/ChNC nanocomposites showed higher char residues at 600 °C compared to both PHU/CNC nanocomposites and neat PHU, which is attributed to the inherently high char residue of chitin after pyrolysis.

**Figure 6:**
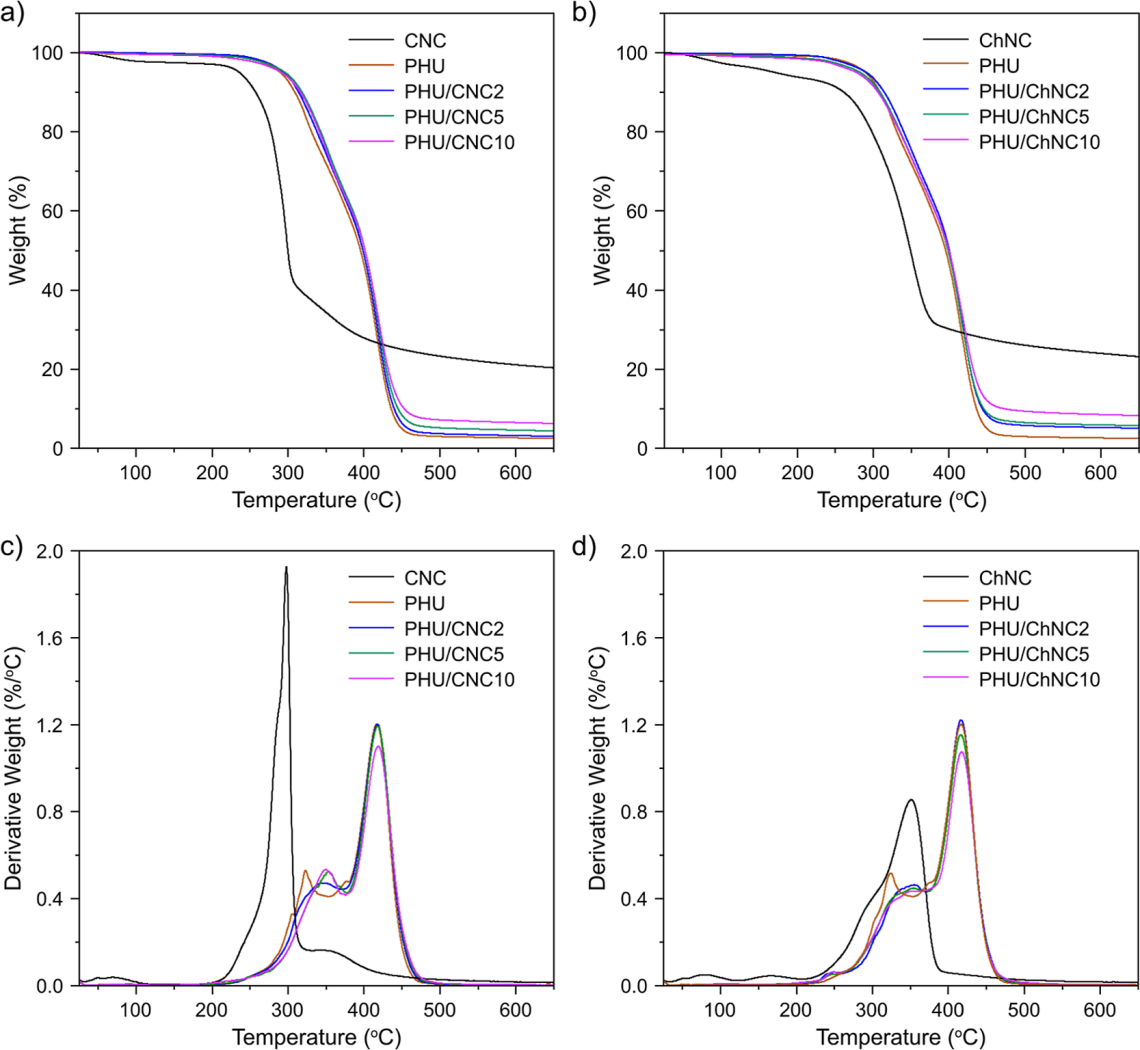
TGA and DTG curves of (a,c) PHU/CNC nanocomposites and (b,d) PHU/ChNC nanocomposites as compared to the neat PHU, CNC, and ChNC.

The mechanical properties of segmented PHU are predominantly influenced by factors including the physical network structure, the structure and molecular weight of the soft segment, the percentage of the hard segment, the cross-linking efficiency, and the degree of hydrogen bonding.^54^ The low tensile strength of the neat segmented PHU indicates a certain degree of phase mixing in these nanophase-separated PHUs, characterized by broad interphase regions.^19^ These interphases arise from hydrogen bonding interactions between the hard and soft segments involving hydroxy and ether groups. These interactions inhibit the segregation of urethane linkages into distinct hard domains.^22,24^ As a result, different from well-phase-separated conventional PUs, the neat segmented PHU exhibited a partial softening of the hard domains due to the presence of hydrogen-bonded soft domains.

The mechanical performance of the PHU/CNC and PHU/ChNC nanocomposites was assessed by a uniaxial tensile test. Figure 7 presents the typical stress–strain curves for both nanocomposites, and their mechanical properties including Young’s modulus, ultimate tensile strength, and strain at break are summarized in Table S2. As shown in Figure 7a, the neat PHU demonstrated distinct transitions marked by a yield point, plastic deformation, and strain hardening leading to an ultimate tensile strength of 0.14 MPa and a strain at break of 1320%, corresponding to the behavior of conventional TPU.^55^ In contrast, both PHU/CNC and PHU/ChNC nanocomposites exhibited a lack of evident plastic deformation and strain hardening following necking. This behavior may stem from the interface interactions between the nanofillers and the PHU matrix, leading to highly constrained regions that restrict polymer chain mobility.^56,57^ Consequently, the polymer chains in the nanocomposites cannot rearrange and align as in the neat segmented PHU, resulting in lower strain at break.^58^ On the other hand, the incorporation of CNCs enhanced the Young’s modulus of the nanocomposites, reaching 1.2 MPa at a CNC content of 10 wt %. This represents a 3-fold increase compared to the neat PHU (0.4 MPa), attributed to the reinforcement effect of well-dispersed CNC nanoparticles within the PHU matrix. The hydrogen bonds between the hydroxy groups on the surfaces of CNC nanoparticles and the urethane groups in the PHU played a crucial role in this enhancement. However, the ultimate tensile strength was not enhanced by the incorporation of CNCs.

**Figure 7:**
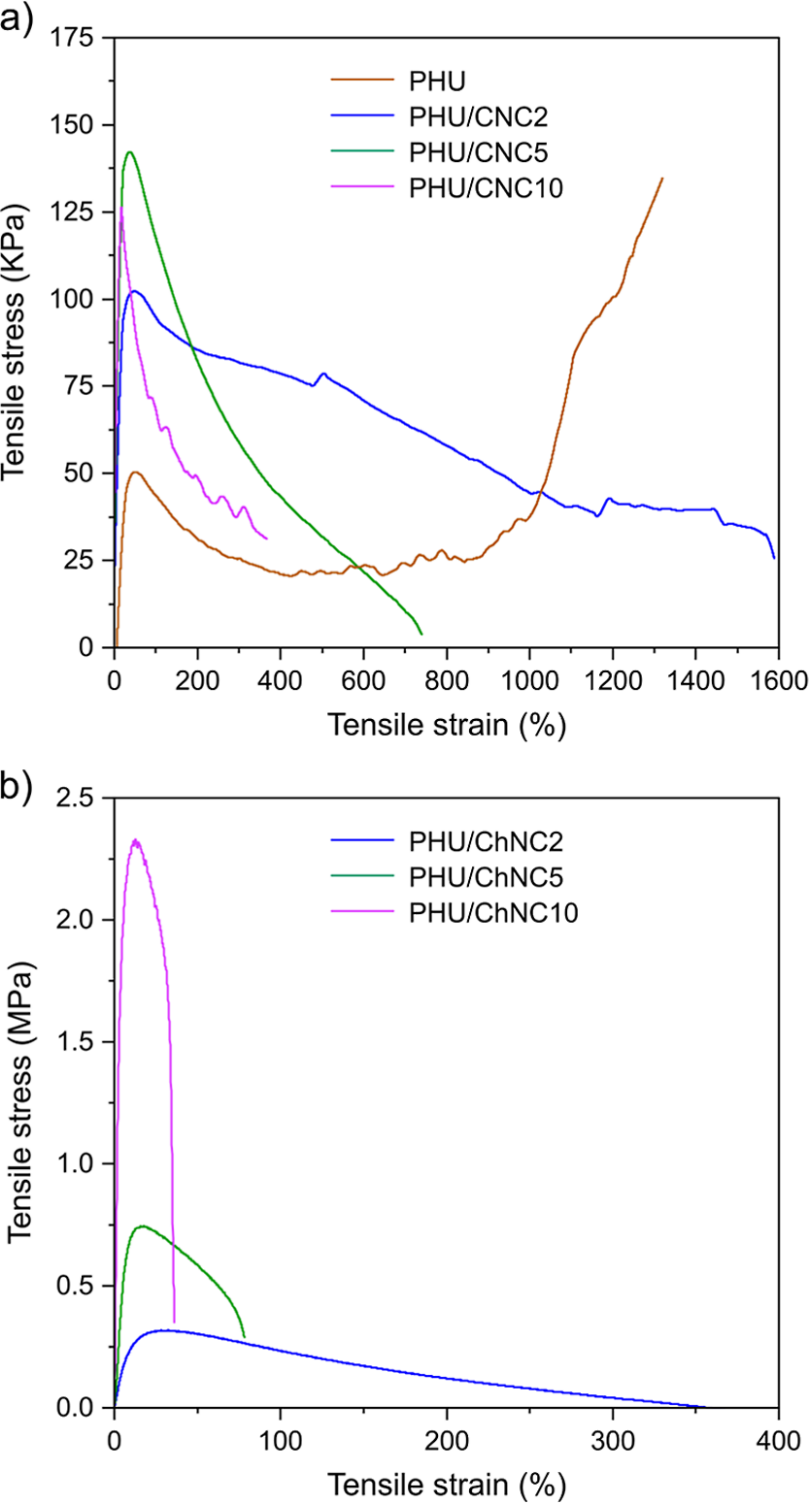
Typical tensile stress–strain curves of (a) the PHU/CNC and (b) PHU/ChNC nanocomposites compared to the neat PHU.

In comparison, the PHU/ChNC nanocomposites exhibited a remarkable enhancement in the mechanical properties (Figure 7b). With the incorporation of 10 wt % ChNC, the Young’s modulus and ultimate tensile strength increased to 58.8 and 2.80 MPa, respectively, representing an outstanding 147-fold improvement in modulus and approximately a 20-fold increase in ultimate tensile strength compared to the neat PHU. These values are comparable to the typical TPUs reported in the literature.^59–61^ These results suggest that the covalent linkages between the partially deacetylated ChNCs and the segmented PHU are the dominant factors influencing the modulus and tensile strength of the nanocomposites, outweighing the contribution of hydrogen bond interactions. The covalently grafted ChNC nanoparticles acted as additional hard segments at the PHU chain ends, contributing to the enhancement of the overall structural strength. The PHU/ChNC nanocomposites showed a progressive increase in Young’s modulus and ultimate tensile strength with higher ChNC contents. However, the strain at break was diminished, further supporting the presence of grafted ChNC nanoparticles.^36,57,62^ The covalent linkages to the PHU polymer created a rigid, cross-linked structure in which the chitin nanocrystals were effectively ″locked″ through their permanent attachment to the hard segments of the segmented PHU polymer. This configuration restricts the reorientation of polymer chains, limiting their ability to undergo significant plastic deformation under a tensile loading. Therefore, while the covalent bonds contribute to increased tensile strength, they significantly reduce the material’s plasticity, leading to lower elongation at break and more brittle behavior in the PHU/ChNC nanocomposites. In addition, ChNCs with a lower degree of deacetylation (∼16%) were prepared via ultrasonication of the chitin extracted from squid and are referred to as ChNC-S. When 10 wt % of ChNC-S was incorporated into the PHU matrix, the resulting nanocomposite (PHU/ChNC-S10) exhibited significantly reduced mechanical performance compared to PHU/ChNC10. Specifically, the ultimate tensile strength and Young’s modulus were measured at 1.20 and 25.6 MPa, respectively (Table S2). These results suggest that the reduced mechanical properties are likely due to the lack of covalent linkages between the ChNCs and the PHU matrix, resulting in weaker interfacial interactions.

The thermomechanical properties of the PHU/CNC and PHU/ChNC nanocomposites were measured by using DMA and are presented in Figure 8. At the lowest temperature of −80 °C, all samples exhibited a fully glassy state, and both nanocomposites displayed a higher storage modulus (*E*′) compared to neat PHU (Figure 8a,b). This difference can be attributed to the presence of rigid CNCs and ChNCs associated with hard segments, which contributes to the enhanced mechanical robustness. As shown in Figure 8c,d, the minor peaks at −66 °C in all loss modulus (*E*″) curves corresponded to the glass transition temperature (*T*_g_) of the PHU soft segment PTMO.^63^ For the neat PHU sample, the glass transition of PTMO soft domains is followed by a broader decrease in *E*′ and *E*″ in the temperature range of −20 to 39 °C with a shoulder peak at 11 °C, corresponding to the *T*_g_ of hard segments. After the flow temperature (*T*_flow_) was 39 °C, the sample lost mechanical robustness and flew out of the DMA grips. *T*_flow_ is related to the nanophase separation and the *T*_g_ of the phase-mixed system.^19^ The broad tan δ peaks observed in the DMA analysis of PHU are indicative of its nanophase-separated morphology, characterized by unusually broad interphase regions and a wide distribution of local compositions. This behavior is attributed to partial phase mixing, where hydrogen bonding interactions between PHU hydroxy groups and PTMO-based soft segments result in a gradual transition between hard and soft domains rather than sharply defined phase boundaries. Similar broad interphase behavior has been reported in previous studies on PHUs and gradient copolymers.^19,24,64^ The PHU/CNC nanocomposites followed the same thermal mechanical behavior as the neat PHU but showed broader tan δ peaks over the temperature range of −20 to 65 °C and higher tan δ values (Figure 8e), suggesting broader interphases in the nanocomposites, as also revealed by SAXS analysis. Particularly, the tan δ value increased significantly between 0 and 20 °C with increasing CNC content, indicating the preferential localization of CNCs within the hard segment. This behavior mirrors the interaction patterns observed in nanocomposites of CNCs with conventional PU as the hard segment is rich in hydrogen-bonding sites that promote strong interactions with CNCs.^65,66^ The PHU/ChNC nanocomposites showed even broader tan δ peaks over the temperature range of −20 to 80 °C but lower tan δ values (Figure 8f), indicating a nanophase-separated system with increased material stiffness owing to the urethane bonds between partially deacetylated ChNC and the hard segments of PHU.

**Figure 8:**
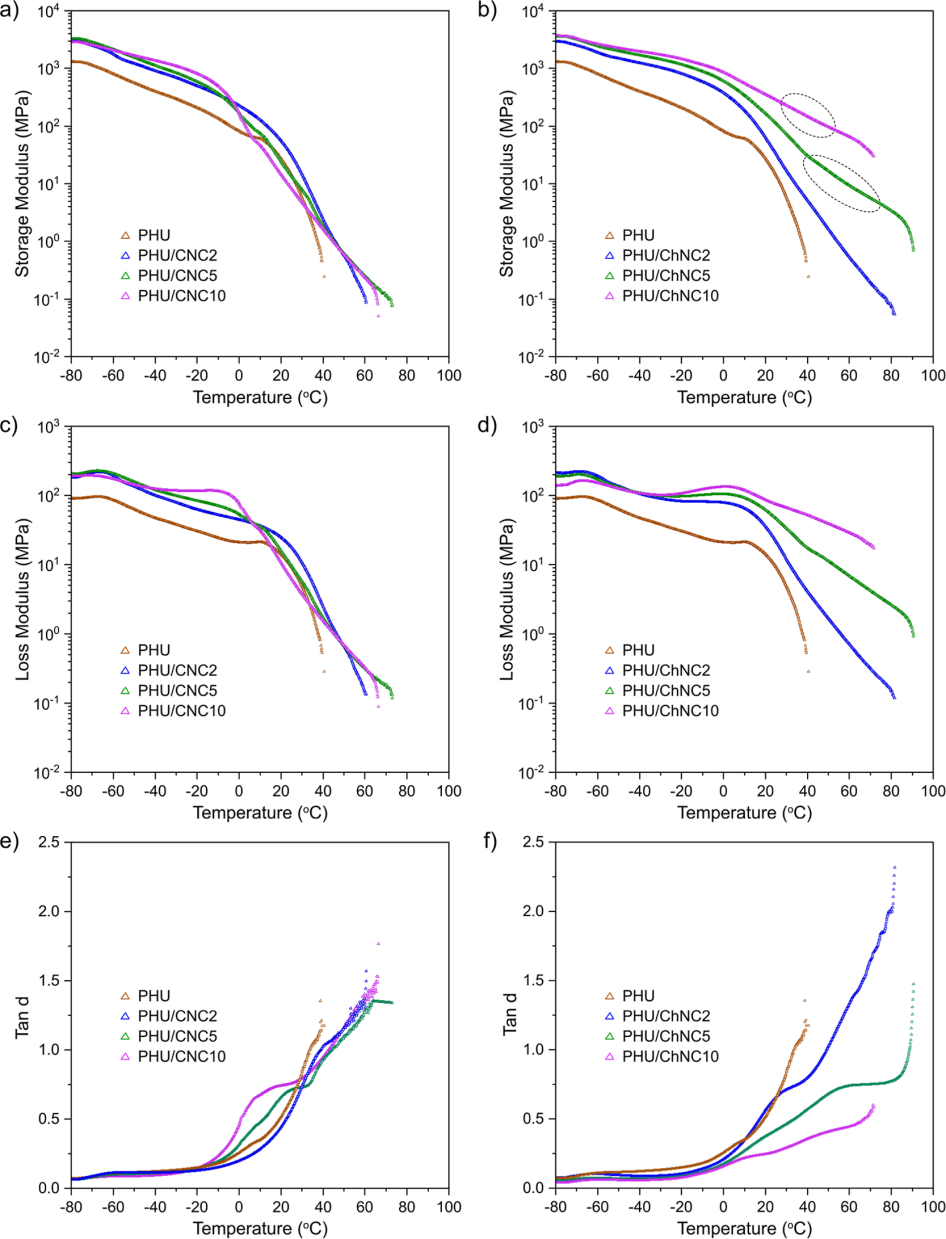
Representative DMA storage modulus (a,b), loss modulus (c,d), and tan δ (e,f) profiles of the PHU/CNC and PHU/ChNC nanocomposites compared to the neat PHU.

Notably, none of the samples exhibited a quasi-rubbery plateau region characteristic of conventional TPU. This suggests a certain degree of phase mixing within the segmented PHU, leading to the formation of broad interphases. Phase mixing in the PHU is facilitated by the ability of hydroxy groups in the hard segments to form hydrogen bonds with the ether oxygen in the PTMO soft segments.^22,24^ The incorporation of CNC and ChNC nanoparticles further promoted phase mixing at higher loadings owing to the increased presence of hydroxy groups capable of forming hydrogen bonds with the segmented PHU. This was evident by the higher *T*_flow_ temperatures of the PHU/CNC (∼65 °C) and PHU/ChNC (∼80 °C) nanocomposites compared to the neat PHU.

The anticipated formation of a rigid CNC network above their percolation threshold (4 wt %, calculated based on aspect ratio^67^) was not observed in the PHU/CNC5 and PHU/CNC10 samples as they showed the same storage modulus as the neat PHU in the rubbery state. The absence of a rigid, continuous CNC network in these samples is attributed to the strong association of CNCs with the hard segments of the PHU matrix driven by hydrogen bonding and polar interactions. Without long-range CNC–CNC interactions from a percolated network, the dynamic mechanical response in the rubbery region was dominated by the PHU matrix. In contrast, although the percolating ChNC network was also not found in the PHU/ChNC nanocomposites, the presence of a significantly enhanced storage modulus region (Figure 8b) following the *T*_g_ of the hard segments is attributed to the stiffening of hard segments with covalently grafted ChNCs and the closer packing of smaller soft and hard domains as revealed by SAXS analysis. Particularly, the *E*′ value of the PHU/ChNC10 sample in the rubbery state was 3 orders of magnitude higher than that of the PHU/CNC nanocomposites.

## 4 Conclusions

In summary, nanocomposites of segmented thermoplastic PHU incorporated with low amounts of CNCs and ChNCs as the reinforcing nanofillers were successfully synthesized and demonstrated enhanced thermal stability, modulus, and ultimate tensile strength. The incorporation of CNCs hindered the onset of thermal degradation of PHU through hydrogen bonding with the hard domains, while the partially deacetylated ChNCs were covalently linked to the hard segments of the segmented PHU, contributing to the significantly increased stiffness and strength of the nanocomposites. Particularly, the PHU/ChNC nanocomposite with 10 wt % ChNC achieved a Young’s modulus of 58.82 MPa and a tensile strength of 2.80 MPa, outperforming the PHU/CNC nanocomposites and the neat PHU. These findings emphasize the crucial role of interfacial covalent bonding between nanofillers and the polymer matrix in enhancing the structural and mechanical properties of PHU nanocomposites, highlighting the advantages of introducing reactive amine groups on the surface of nanofillers.

## Supplementary Material



## Notes

Photographs, ^1^H NMR spectra, and SEC chromatograms of the segmented PHU samples; AFM height images, width and length distributions, and XRD pattern of CNC and ChNC; FTIR spectra of ChNC and partially deacetylated ChNCs; HR MAS ^1^H NMR spectra of partially deacetylated ChNCs; AFM inphase images with hard domain size distribution histograms; and summary of the thermal and mechanical properties of the nanocomposites (PDF)
