# Supplementary material for: Synthesis, Thermal and Mechanical Properties of Nonisocyanate Thermoplastic Polyhydroxyurethane Nanocomposites with Cellulose Nanocrystals and Chitin Nanocrystals
Source: Biomacromolecules. 2025 May 9;26(6):3481–94. doi: 10.1021/acs.biomac.5c00113 (PMC12152971; doi:10.1021/acs.biomac.5c00113)
Supplement: Supplementary file 1 [file bm5c00113_si_001.pdf]

Supporting Information

Synthesis, Thermal and Mechanical Properties of Nonisocyanate Thermoplastic Polyhydroxyurethane Nanocomposites with Cellulose Nanocrystals and Chitin Nanocrystals

Pavithra M. Wijeratne, [†, ‡] *Connie Ocando,* [‡] *Bruno Grignard,* [§] *Lars A. Berglund,* [⊥] *Jean-Marie Raquez,* [‡] *and Qi Zhou* [*, †]

[†]Division of Glycoscience, Department of Chemistry, School of Engineering Sciences in Chemistry, Biotechnology and Health, KTH Royal Institute of Technology, AlbaNova University Centre, 106 91, Stockholm, Sweden

[‡]Laboratory of Polymeric and Composite Materials, Department of Chemistry, Faculty of Science, University of Mons, 7000 Mons, Belgium

[§]Center for Education and Research on Macromolecules (CERM), CESAM Research Unit, University of Liege, Sart-Tilman B6a, 4000 Liege, Belgium

[⊥]Department of Fiber and Polymer Technology, KTH Royal Institute of Technology, 100 44 Stockholm, Sweden

* E-mail: qi@kth.se

Supporting Information contains 26 pages including 24 Figures and 2 Tables.

1. Characterization of the Segmented PHUs.

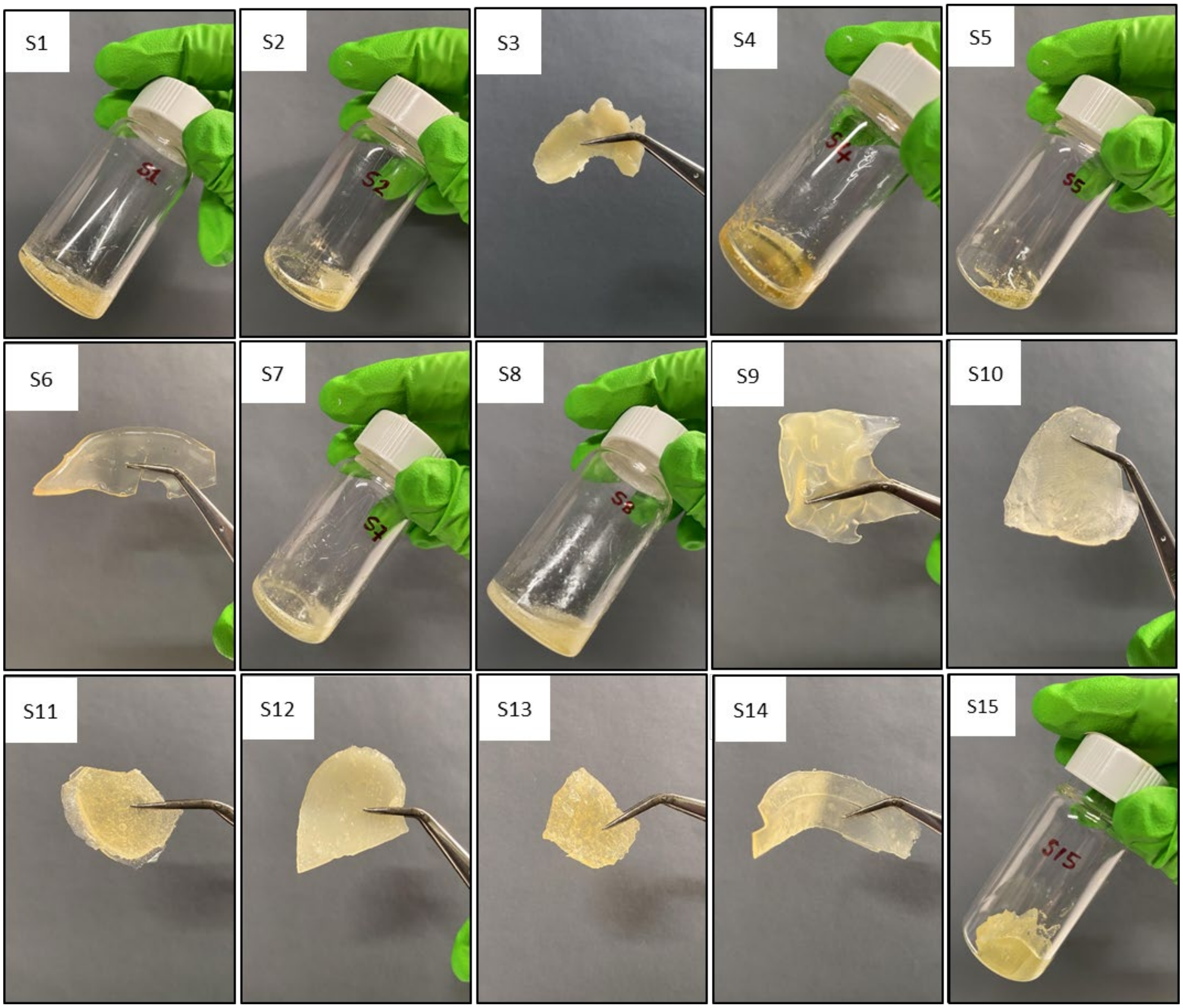

Figure S1. Photographs of the segmented PHU samples.

(S1: VABC/ED-600/m-XDA, S2: VABC/ED-900/m-XDA, S3: VABC/PPGDA/m-XDA, S4: VABC/ED-600/NORB, S5: VABC/ED-900/NORB, S6: VABC/PPGDA/NORB, S7: VABC/ED-600/Dytek-A, S8: VABC/ED-900/Dytek-A, S9: VABC/PPGDA/Dytek-A, S10: VABC/PTMODA/NORB, S11: VABC/PEGDA/NORB, S12: VABC/PTMODA/Dytek-A, S13: VABC/PEGDA/Dytek-A, S14: BPADC/PTMODA/NORB, S15: BPADC/PEGDA/NORB)

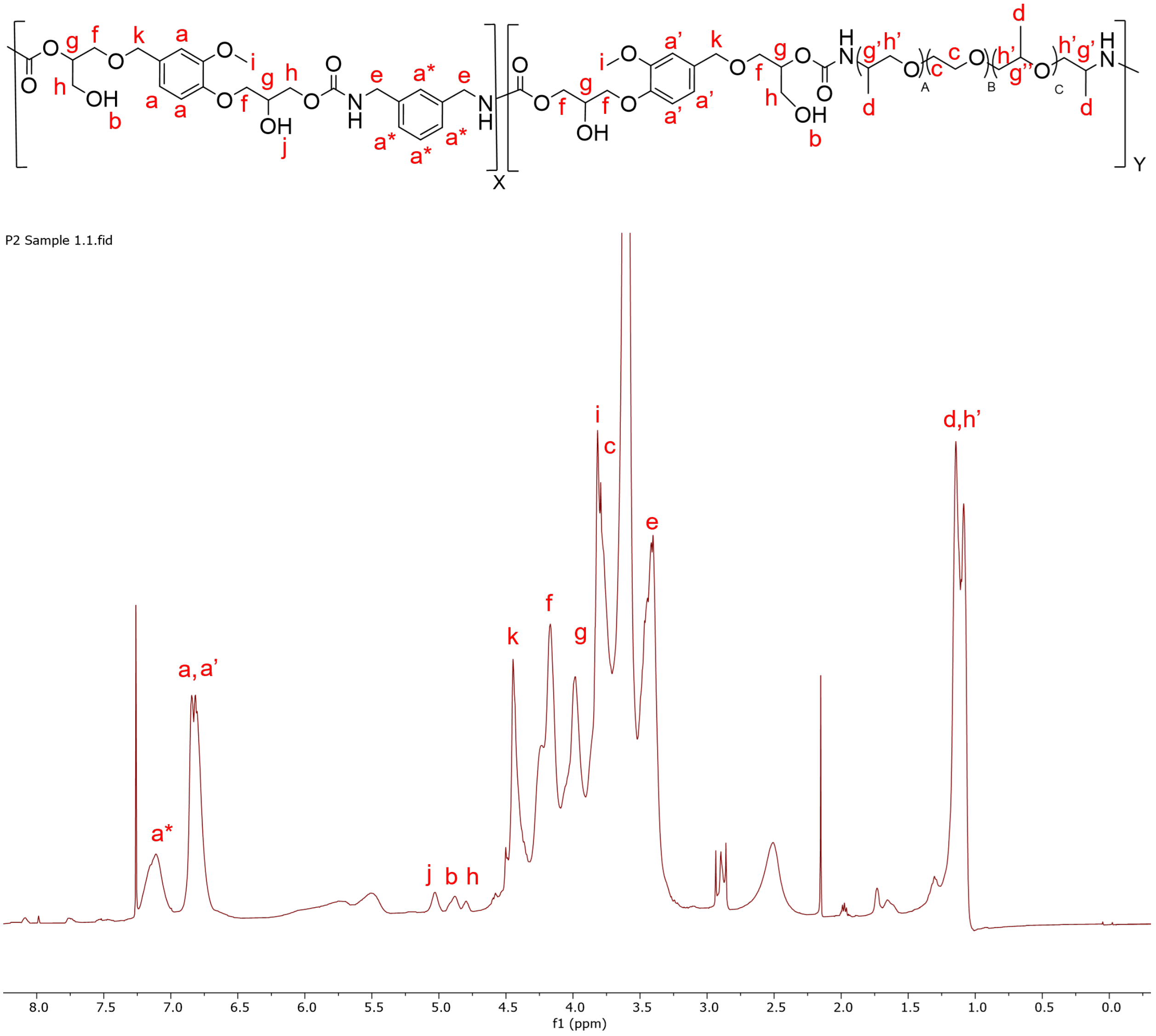

Figure S2. ^{1}H NMR Spectrum of the VABC/ED-600/m-XDA PHU sample in $CDCl_3$.

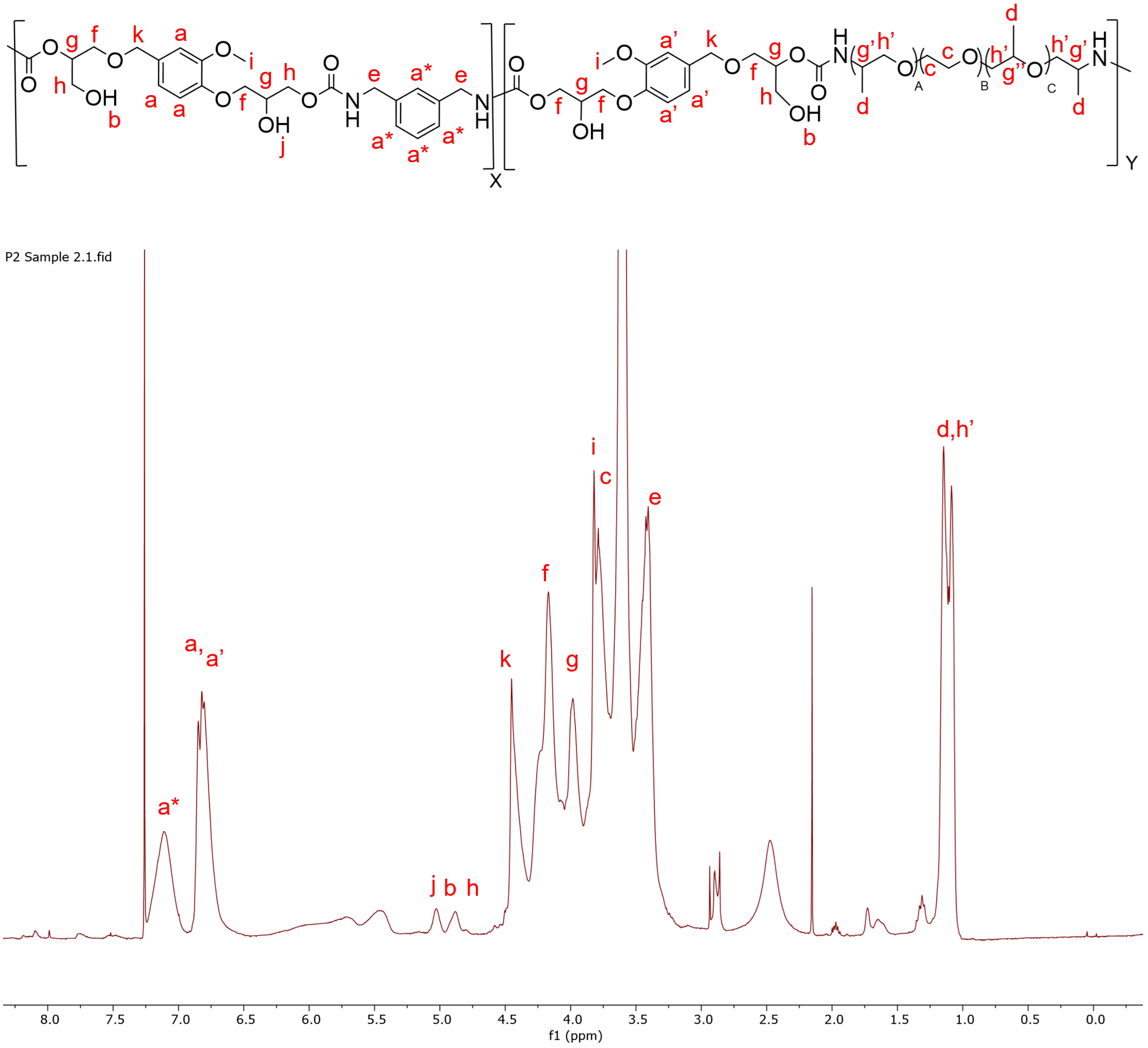

Figure S3. ^{1}H NMR Spectrum of VABC/ED-900/m-XDA PHU sample in $CDCl_3$.

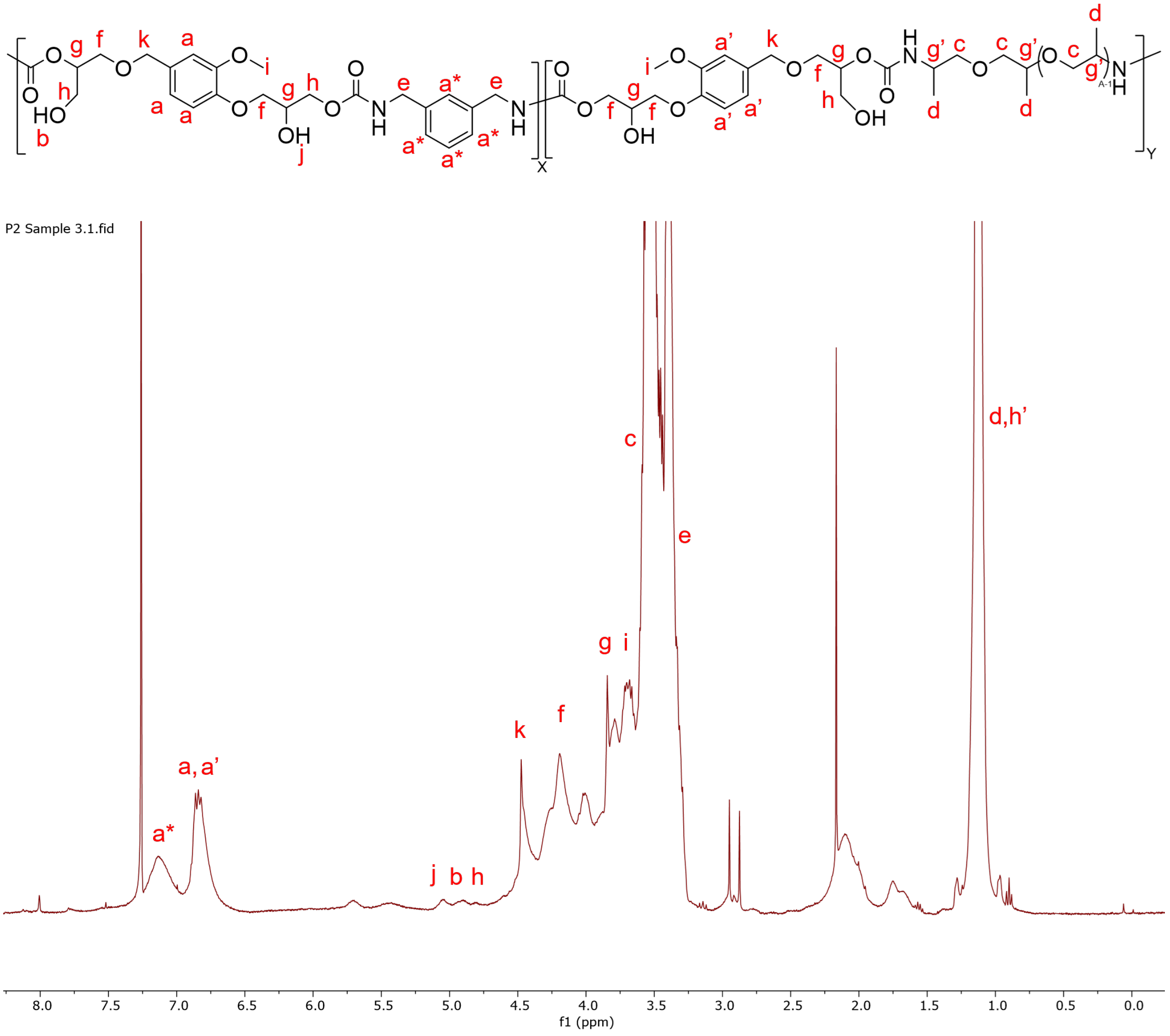

Figure S4. ^{1}H NMR Spectrum of VABC/PPGDA/m-XDA PHU sample in $CDCl_3$.

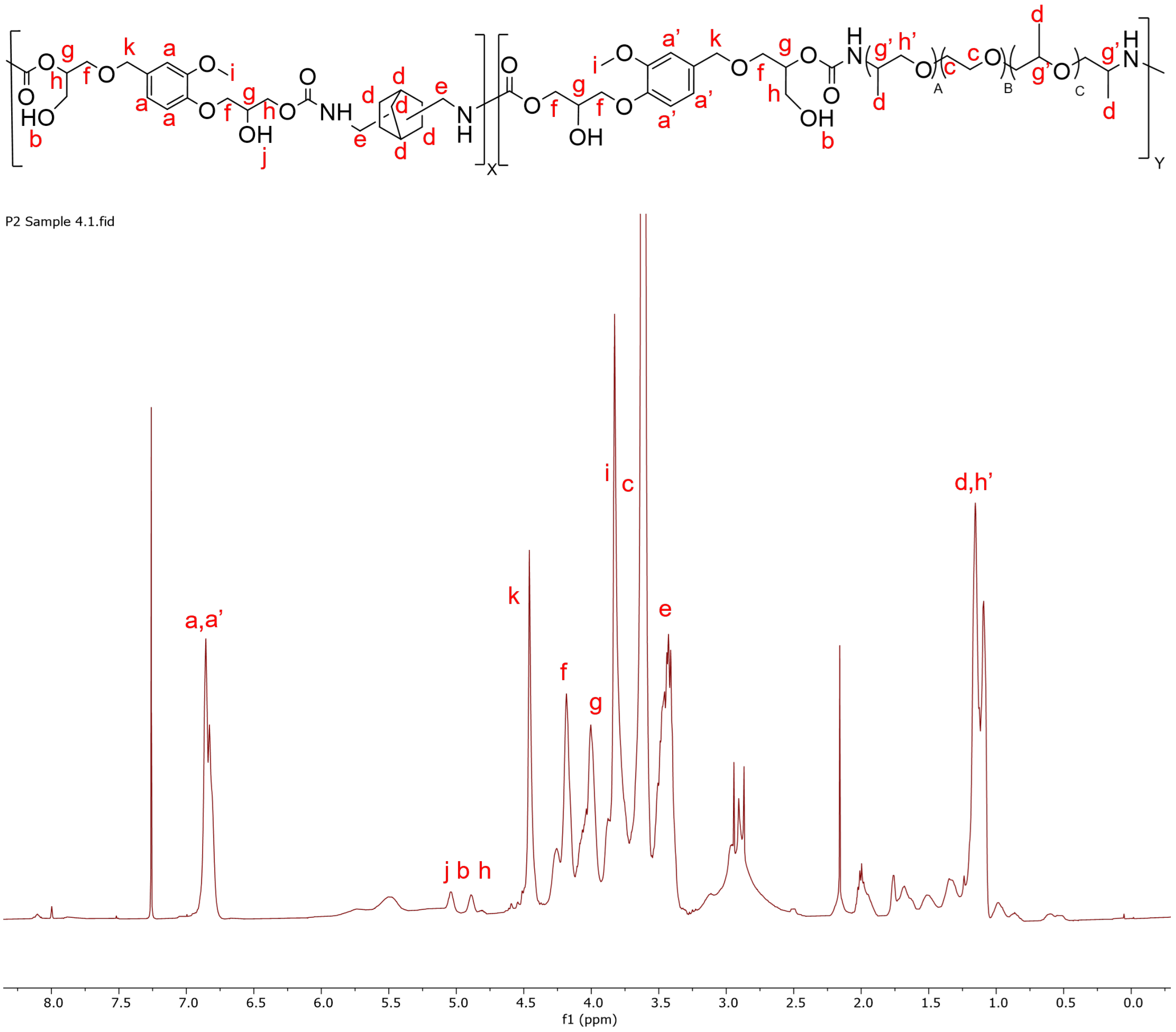

Figure S5. ^{1}H NMR Spectrum of VABC/ED-600/NORB PHUsample in $CDCl_3$.

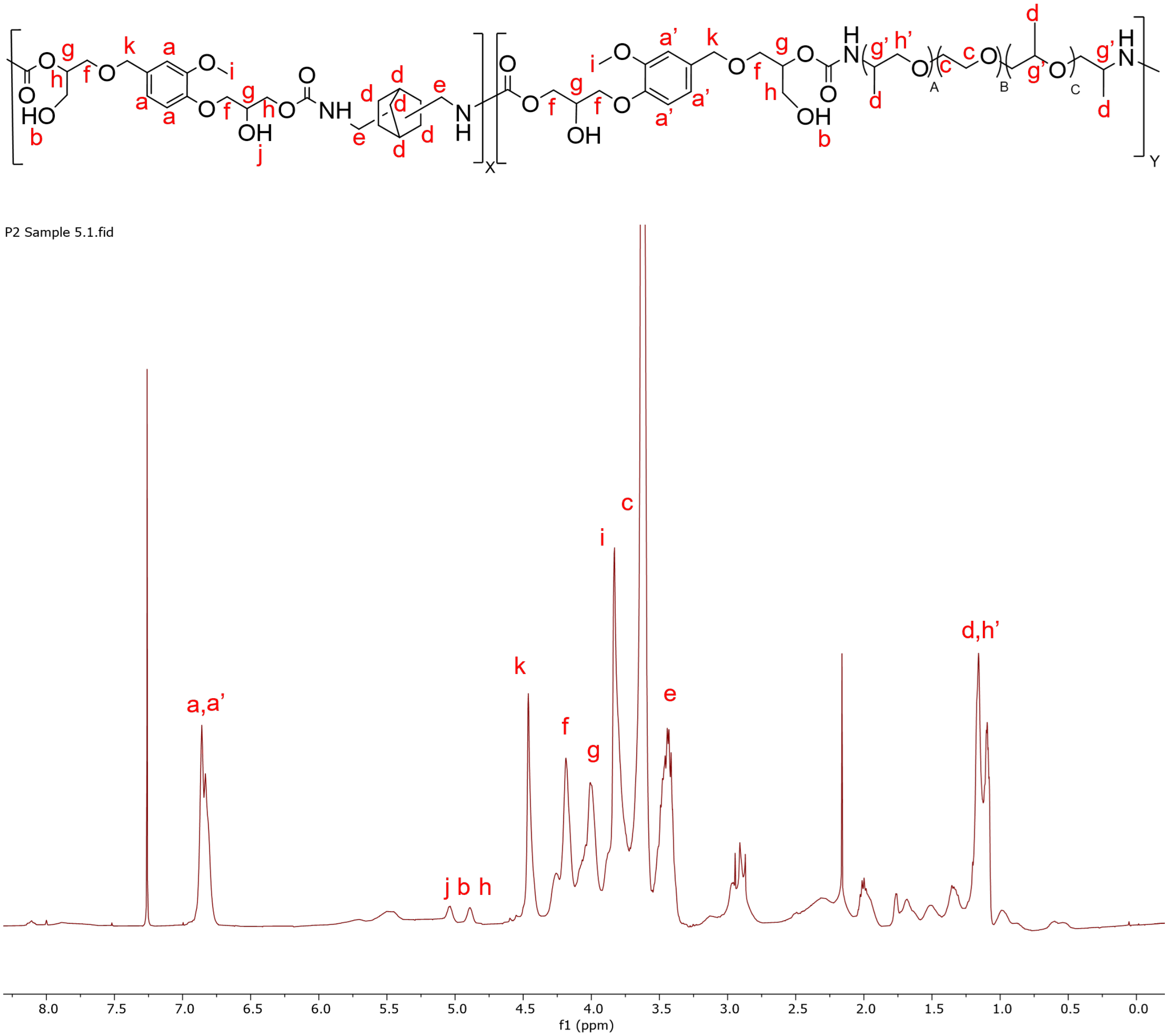

Figure S6. ^{1}H NMR Spectrum of VABC/ED-900/NORB PHU sample in $CDCl_3$.

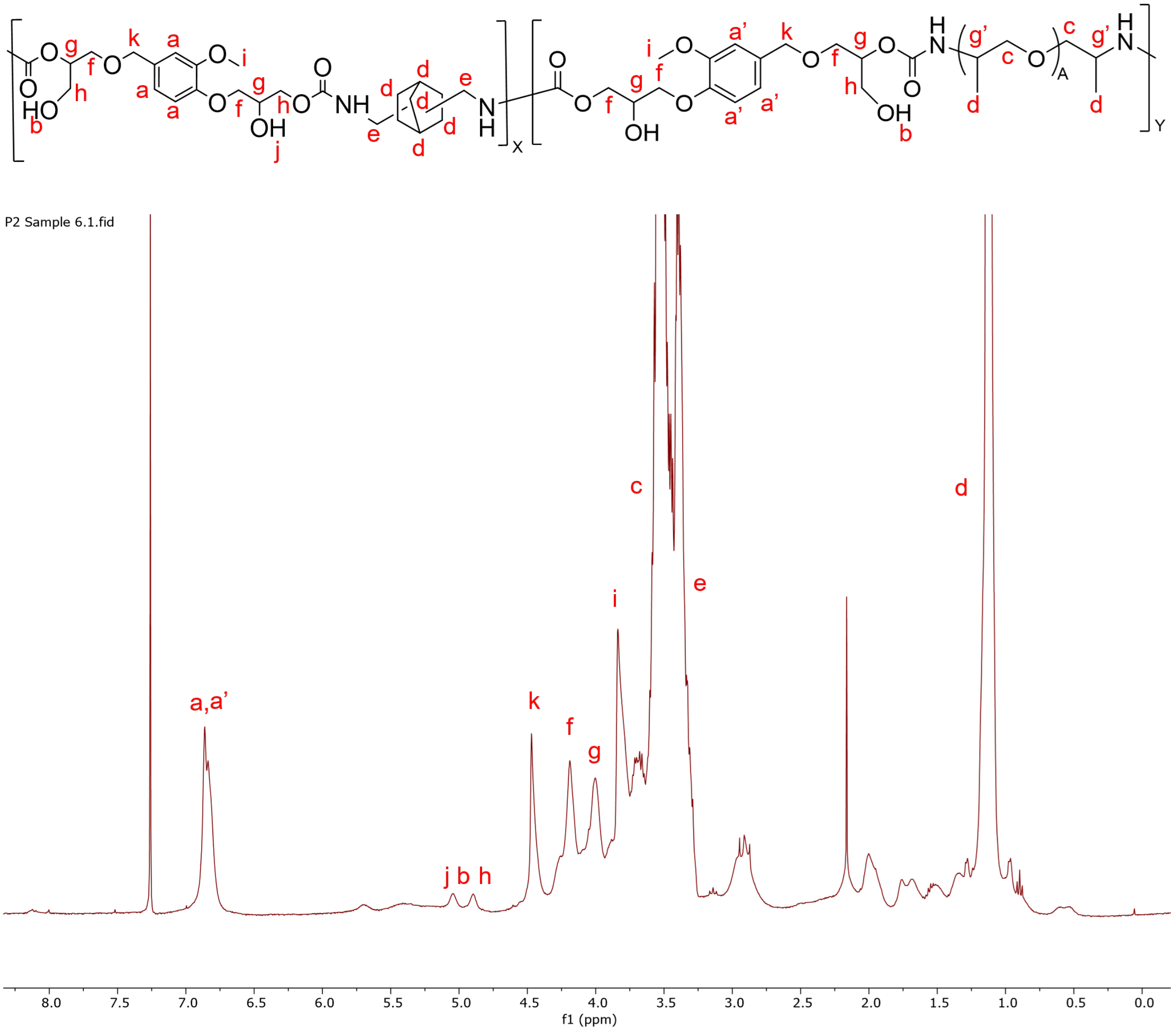

Figure S7. ^{1}H NMR Spectrum of VABC/PPGDA/NORB PHU sample in $CDCl_3$.

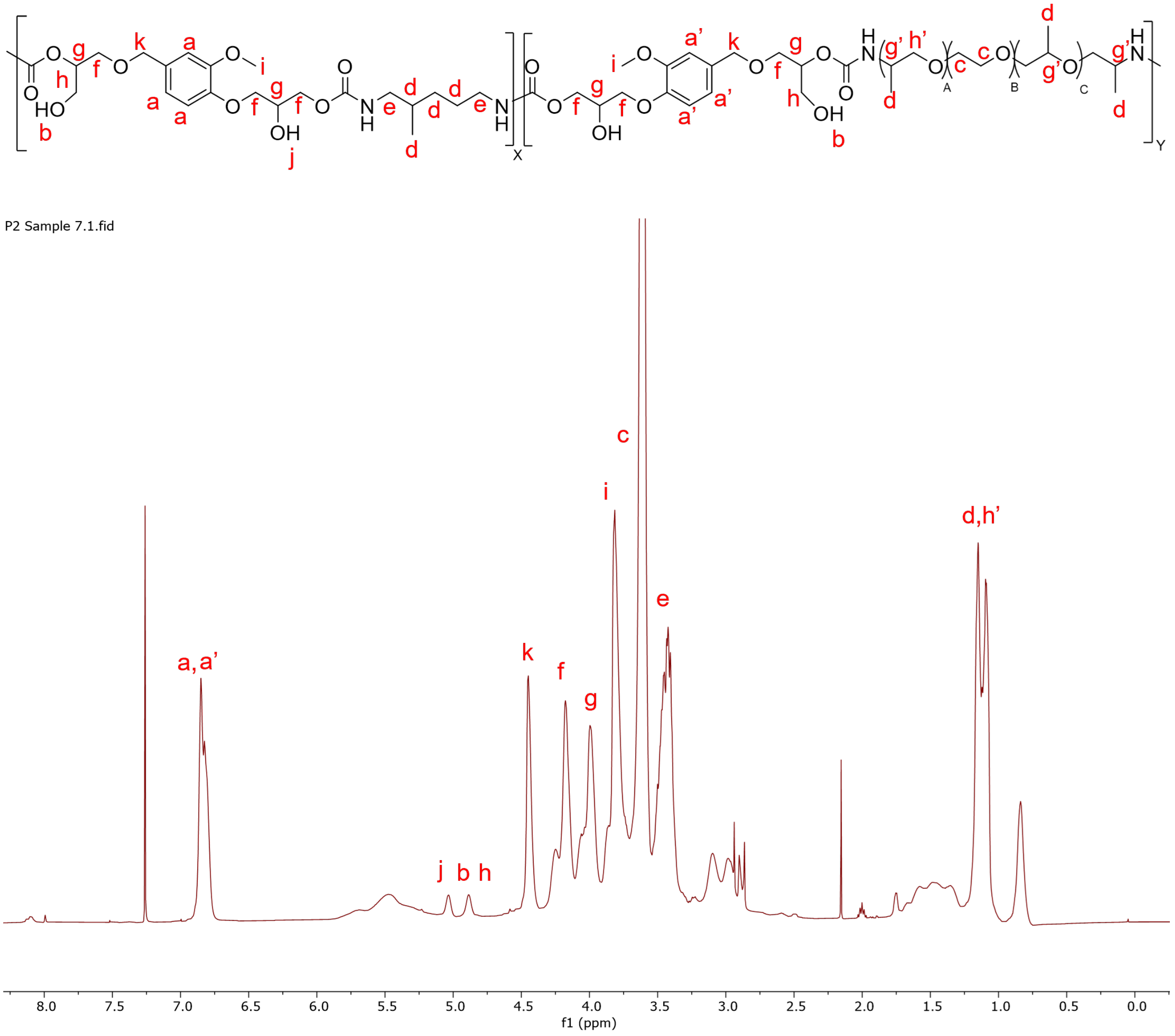

Figure S8. ^{1}H NMR Spectrum of VABC/ED-600/Dytek-A PHU sample in $CDCl_3$.

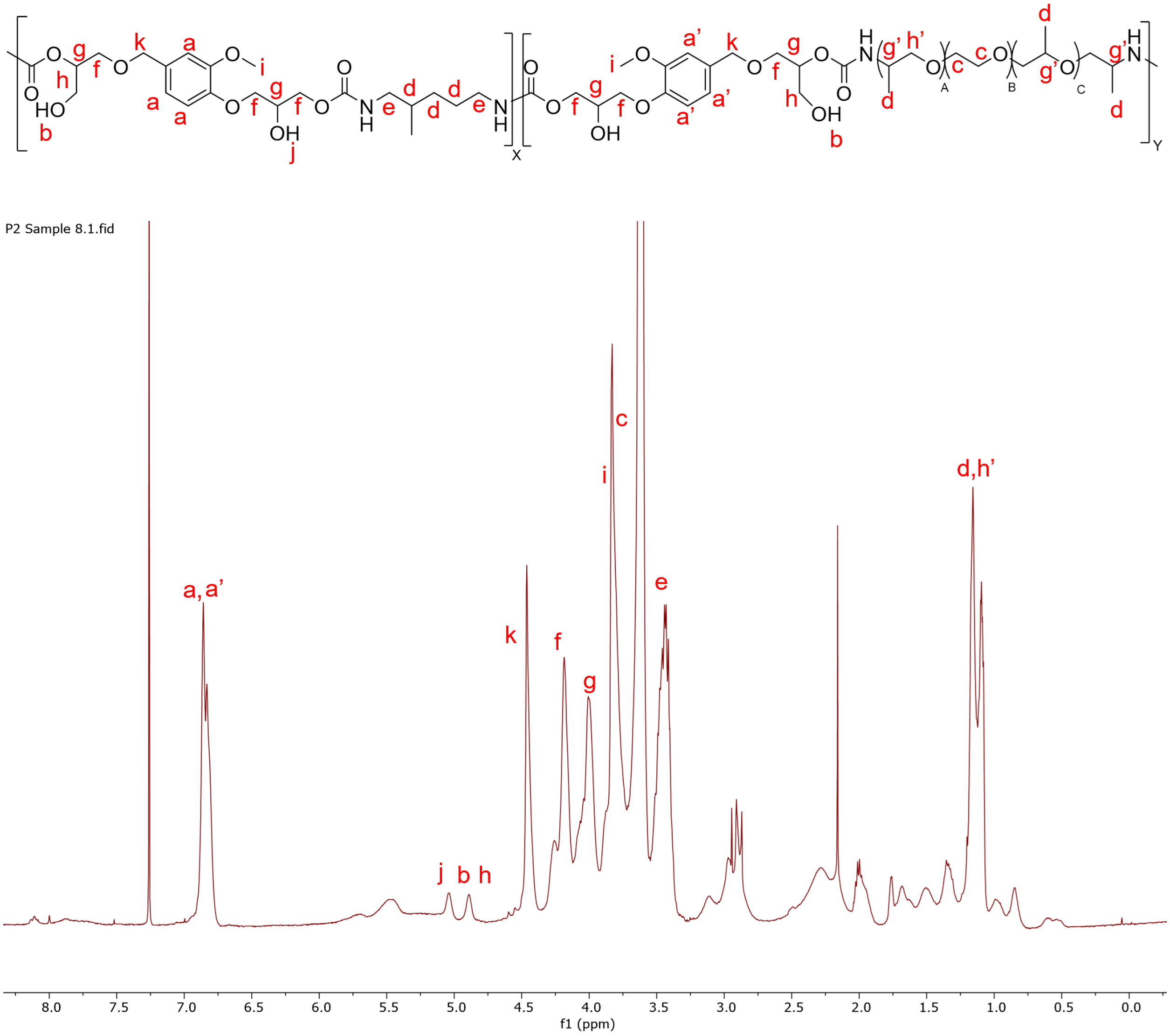

Figure S9. ^{1}H NMR Spectrum of VABC/ED-900/Dytek-A PHU sample in $CDCl_3$.

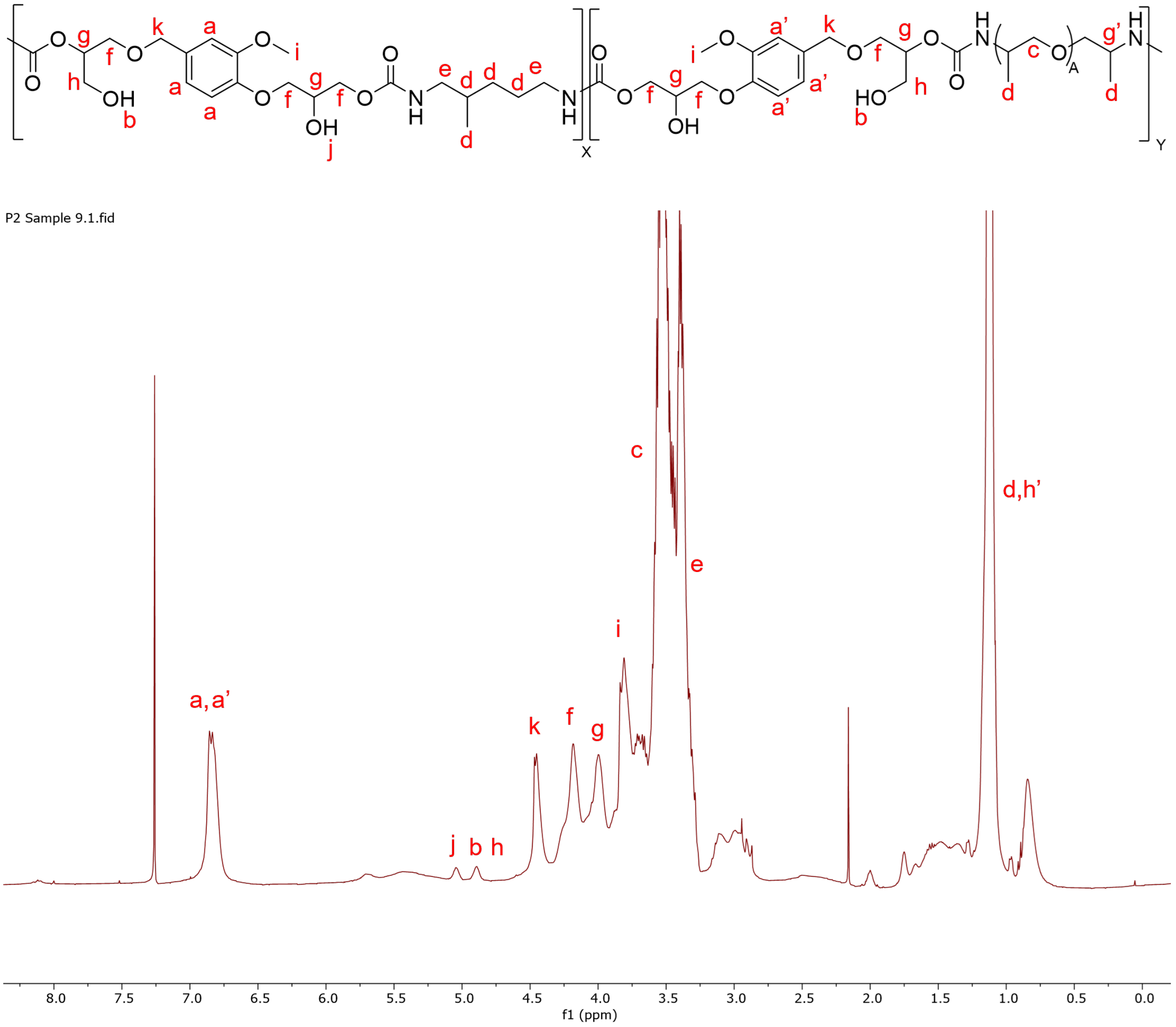

Figure S10. ^{1}H NMR Spectrum of VABC/PPGDA/Dytek-A PHU sample in $CDCl_3$.

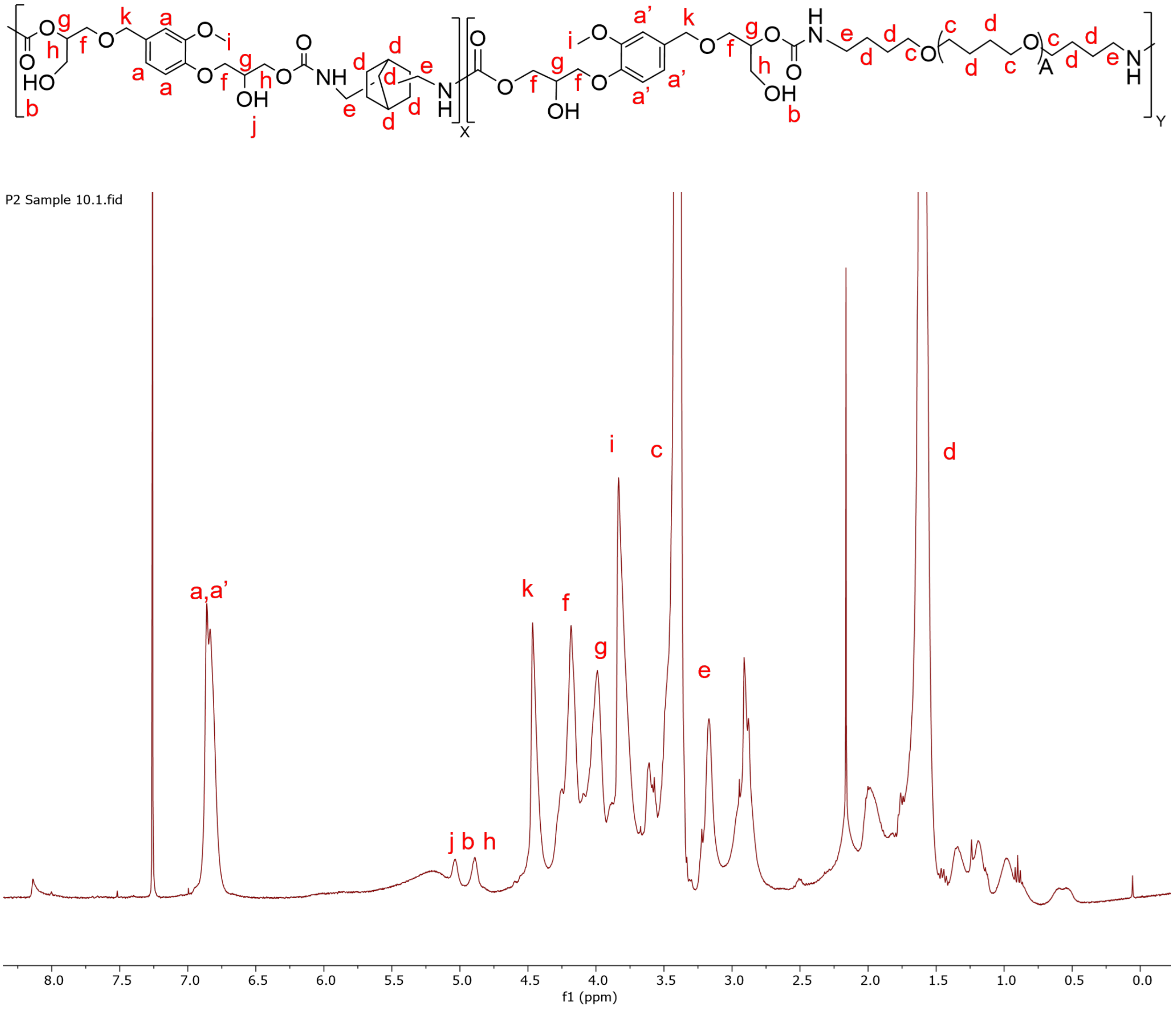

Figure S11. ^{1}H NMR Spectrum of VABC/PTMODA/NORB PHU sample in $CDCl_3$.

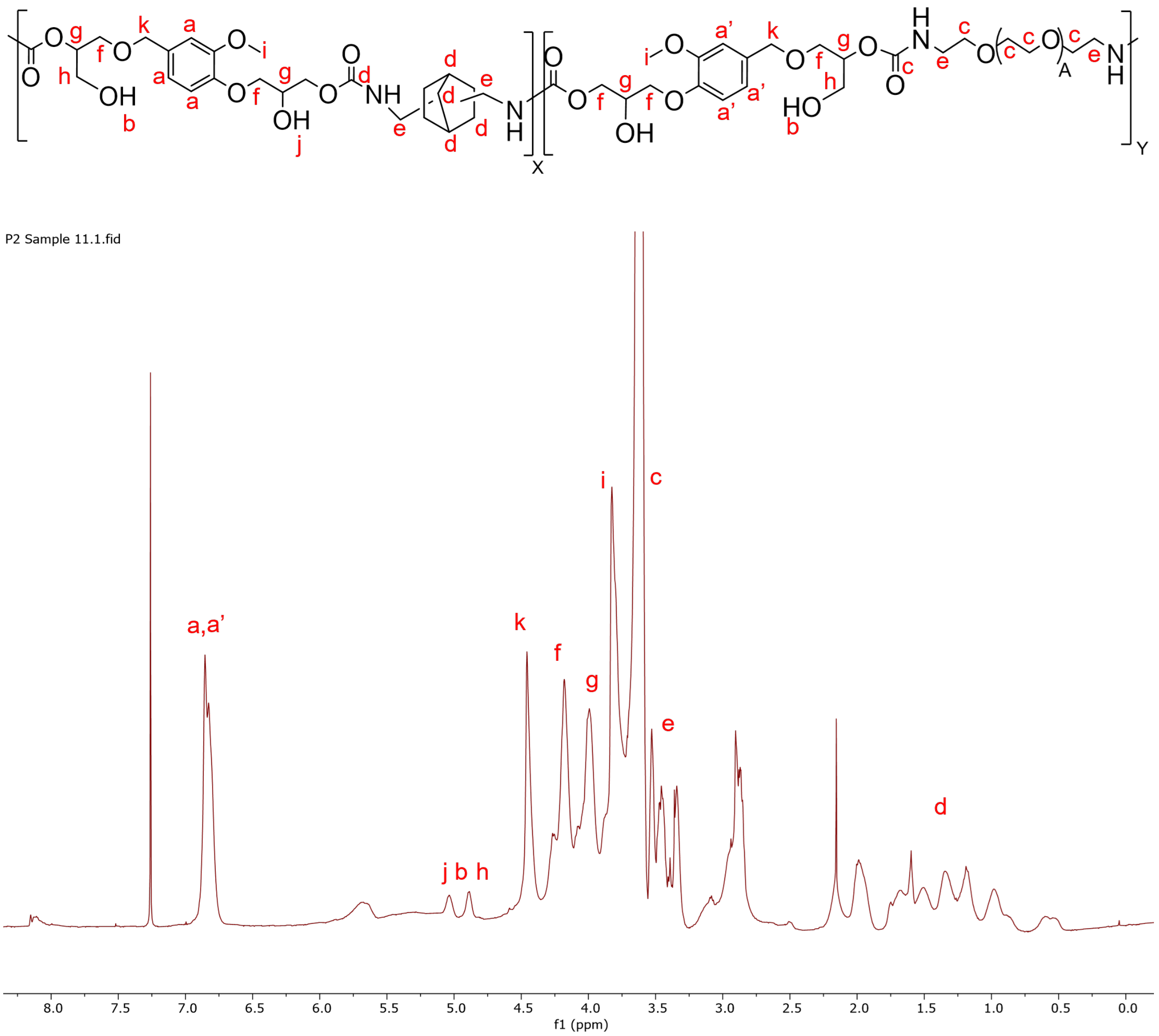

Figure S12. ^{1}H NMR Spectrum of VABC/PEGDA/NORB PHU sample in $CDCl_3$.

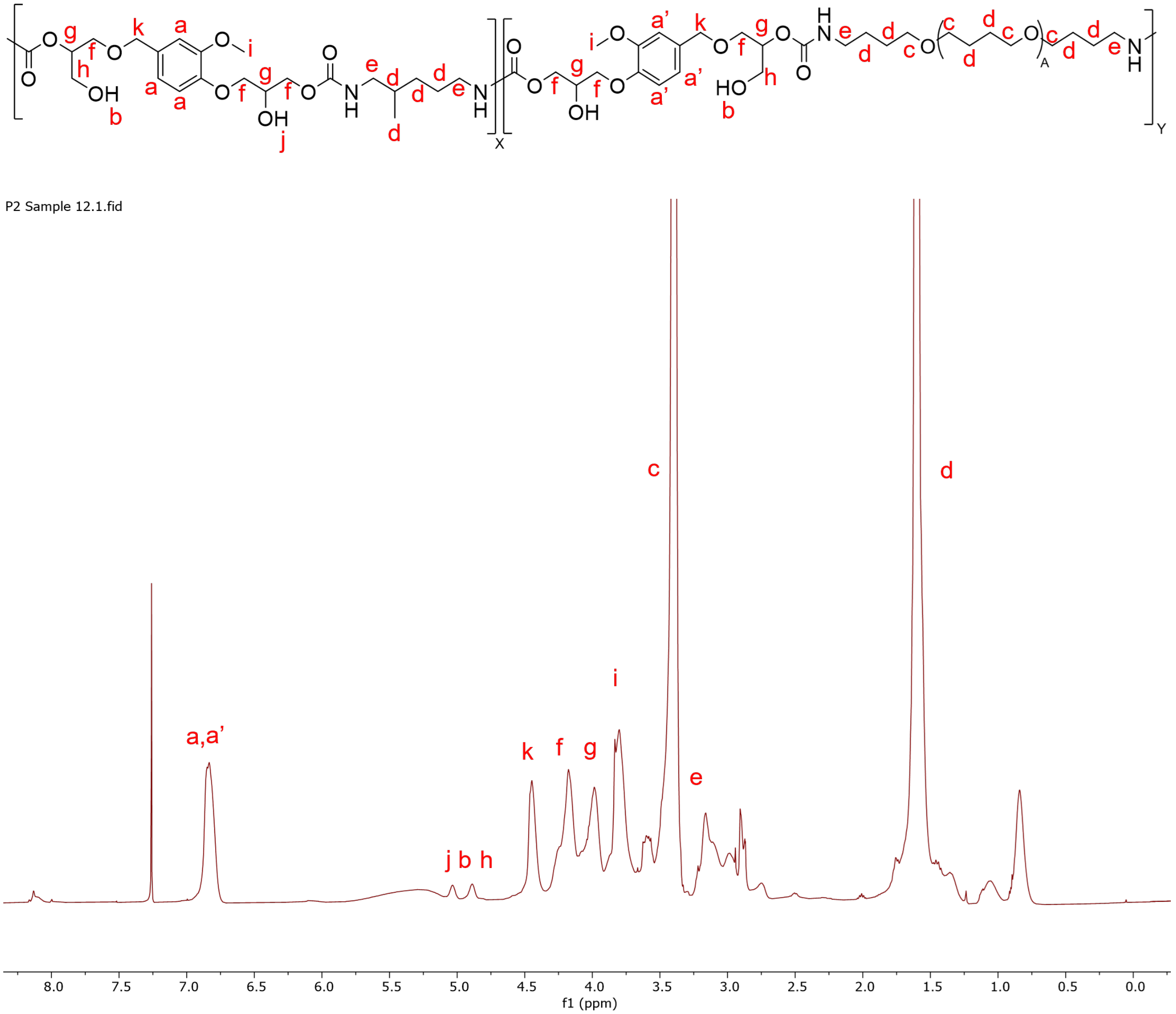

Figure S13. ^{1}H NMR Spectrum of VABC/PTMODA/Dytek-A PHU sample in $CDCl_3$.

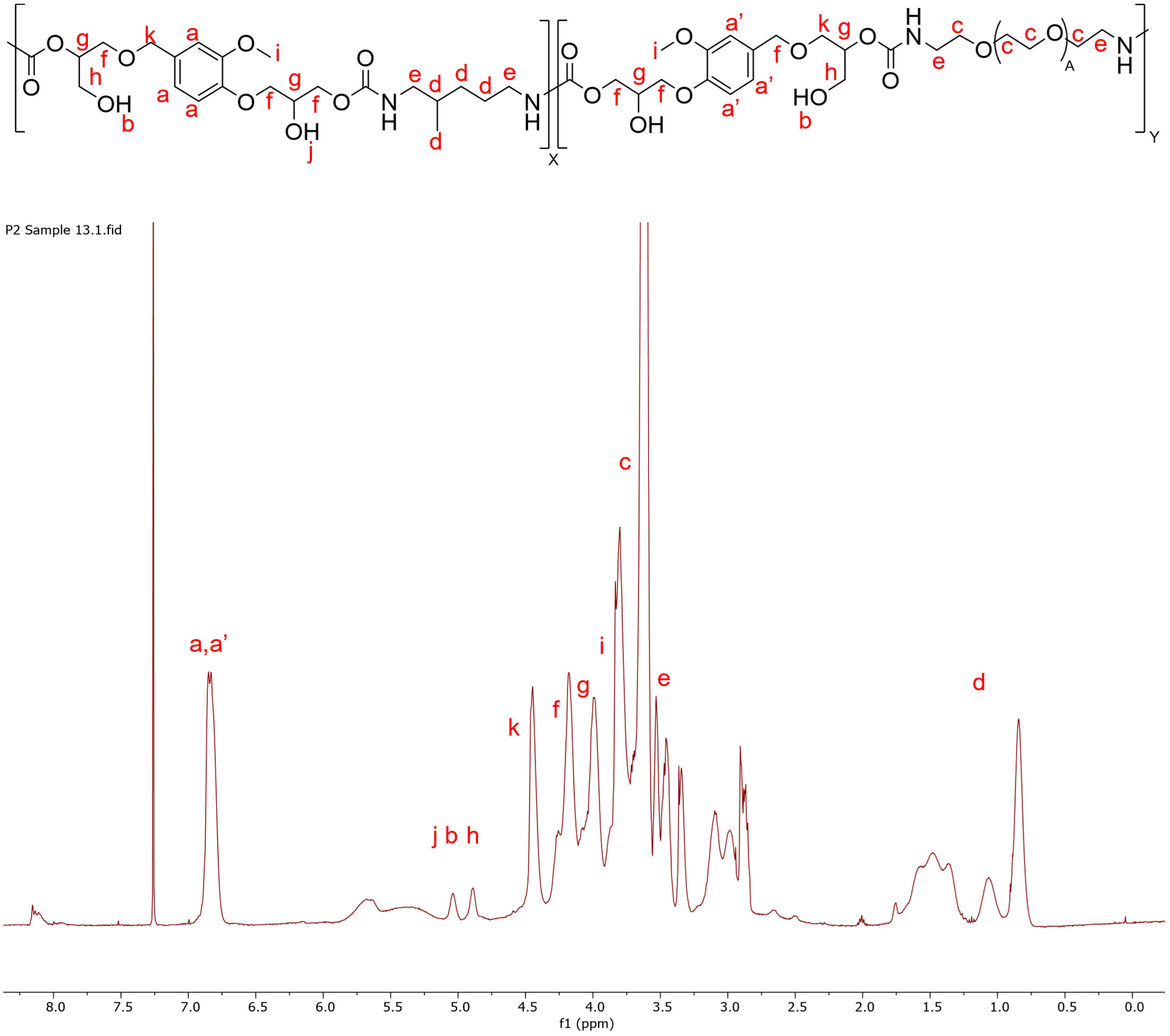

Figure S14. ^{1}H NMR Spectrum of VABC/PEGDA/Dytek-A PHU sample in $CDCl_3$.

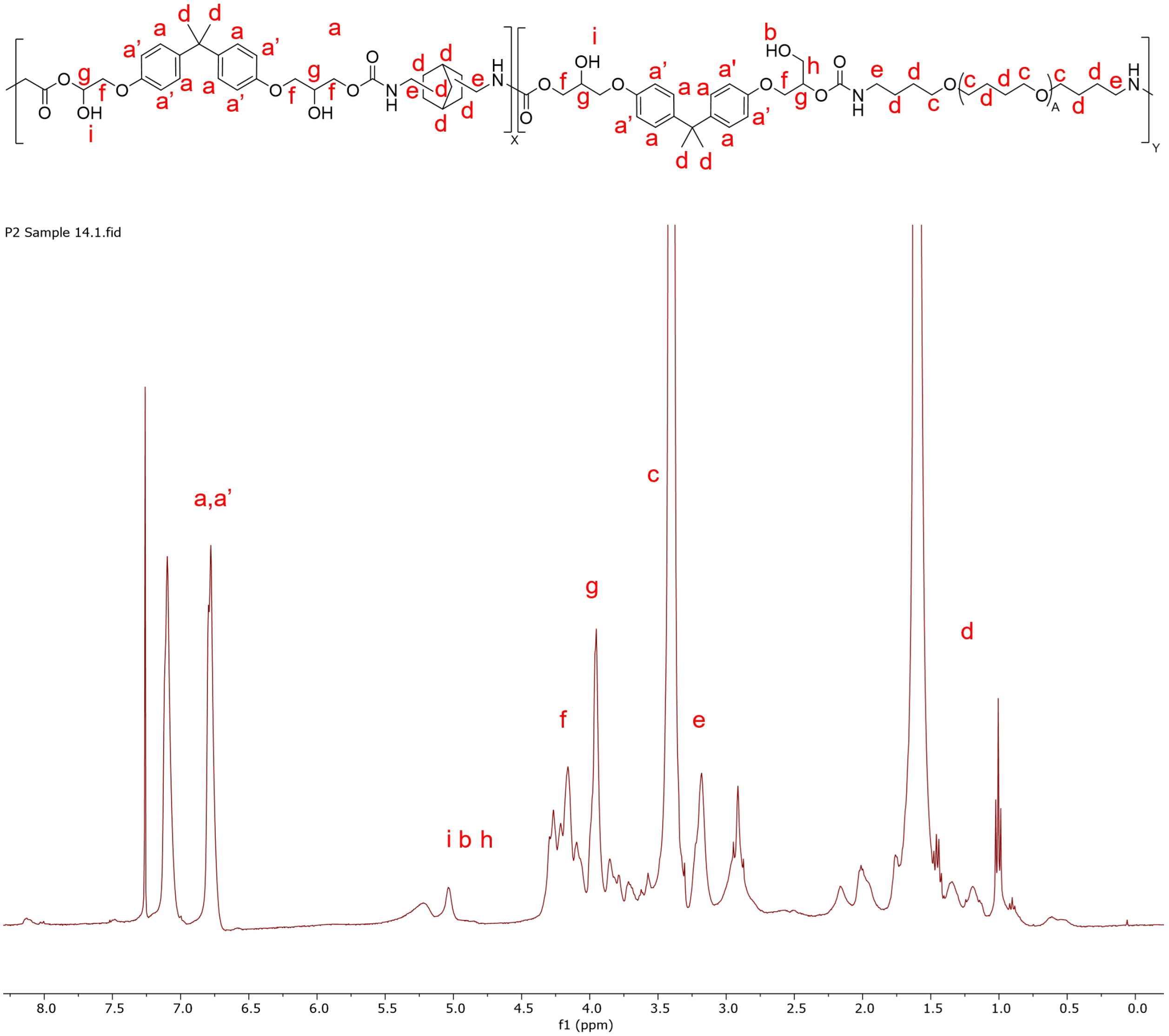

Figure S15. ^{1}H NMR Spectrum of BPADC/PTMODA/NORB PHU sample in $CDCl_3$.

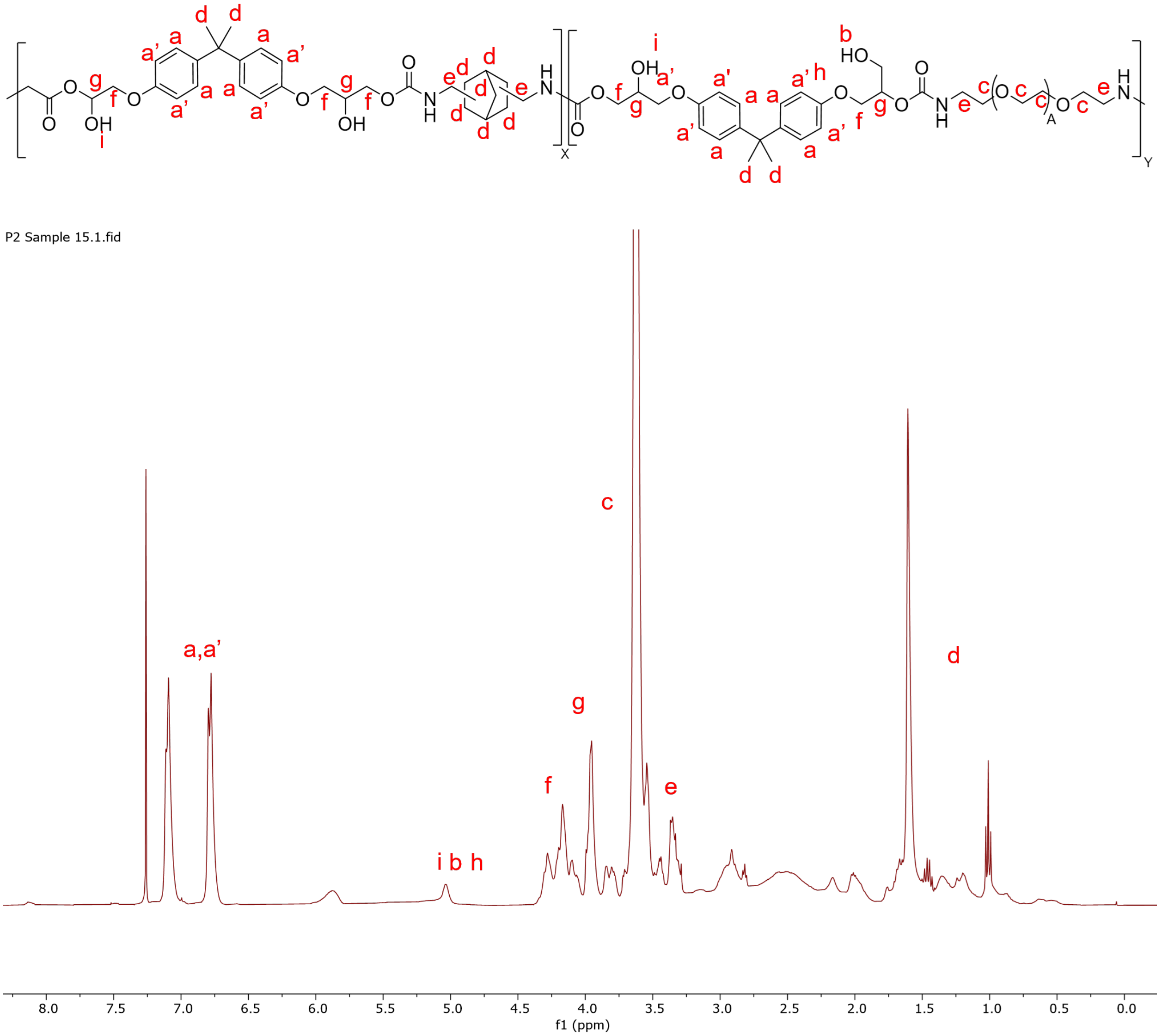

Figure S16. ^{1}H NMR Spectrum of BPADC/PEGDA/NORB PHU sample in $CDCl_3$.

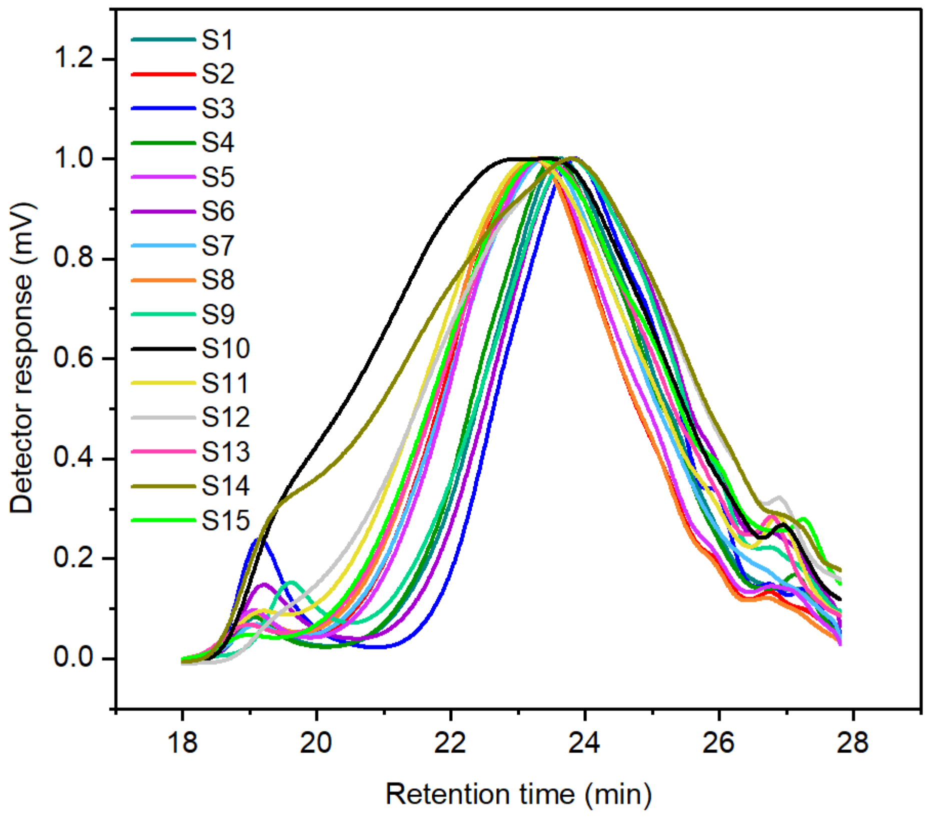

Figure S17. SEC chromatograms of the segmented PHU samples.

(S1: VABC/ED-600/m-XDA, S2: VABC/ED-900/m-XDA, S3: VABC/PPGDA/m-XDA, S4: VABC/ED-600/NORB, S5: VABC/ED-900/NORB, S6: VABC/PPGDA/NORB, S7: VABC/ED-600/Dytek-A, S8: VABC/ED-900/Dytek-A, S9: VABC/PPGDA/Dytek-A, S10: VABC/PTMODA/NORB, S11: VABC/PEGDA/NORB, S12: VABC/PTMODA/Dytek-A, S13: VABC/PEGDA/Dytek-A, S14: BPADC/PTMODA/NORB, S15: BPADC/PEGDA/NORB)

2. Structure of CNCs and ChNCs.

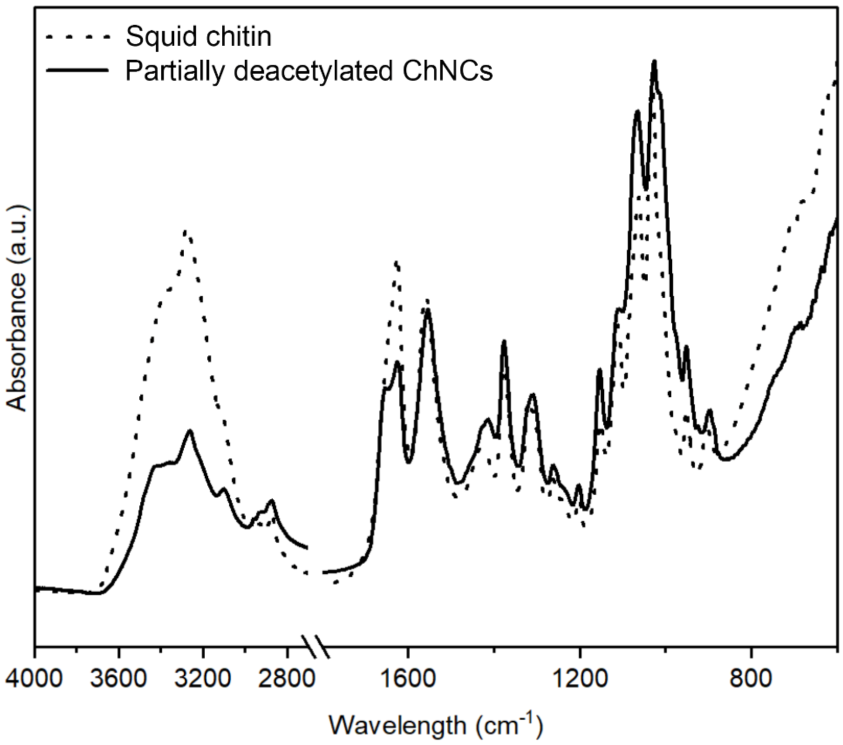

Figure S18. ATR-FTIR spectra of the extracted squid chitin and partially deacetylated chitin nanocrystals (ChNCs).

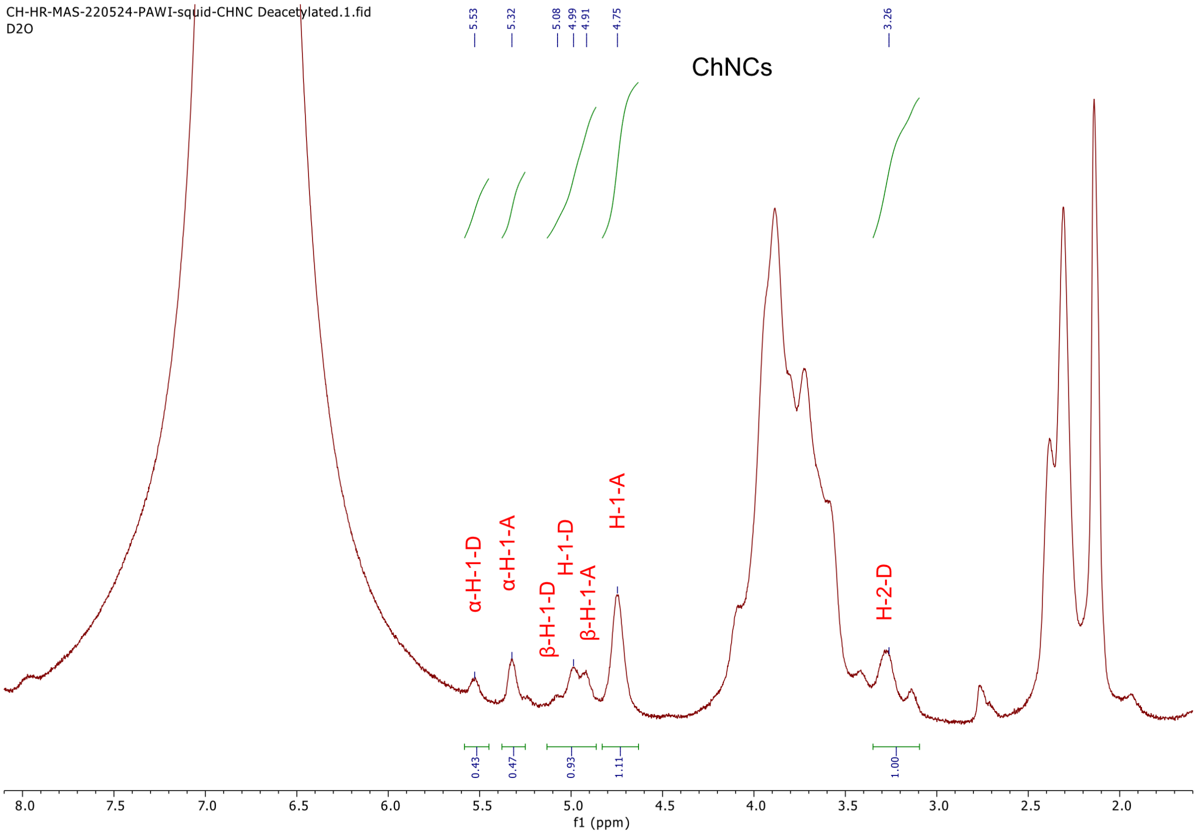

Figure S19. HR MAS ^{1}H NMR spectra of the partially deacetylated ChNCs in 20 wt% DCl solution in D_2O.

The ^{1}H NMR spectrum displays characteristic resonances in the anomeric region corresponding to both acetylated and deacetylated units. The H-1 proton of acetylated α- and β-anomers exhibit signals at 5.32 ppm and 4.91 ppm, respectively. The H-1 proton of internal deacetylated units appears at 4.99 ppm, overlapping with the β-anomeric signal, while the H-1 of internal acetylated units resonates at 4.75 ppm. Additionally, the H-2 signal of internal deacetylated units is observed at 3.26 ppm. For the reducing ends of deacetylated units, the α- and β-anomeric H-1 protons are found at 5.53 ppm and 5.08 ppm, respectively.

The degree of deacetylation (DDA) of chitin was calculated based on the integral of the H-2 resonance of deacetylated units (H-2-D) relative to the total integral of all H-1 protons, including α-H-1-A, α-H-1-D, β-H-1-A, β-H-1-D, H-1-A, and H-1-D. Using the integral values:

$$DDA = [1.00 / (0.43 + 0.47 + 0.93 + 1.11)] \times 100\% = 34\%.$$

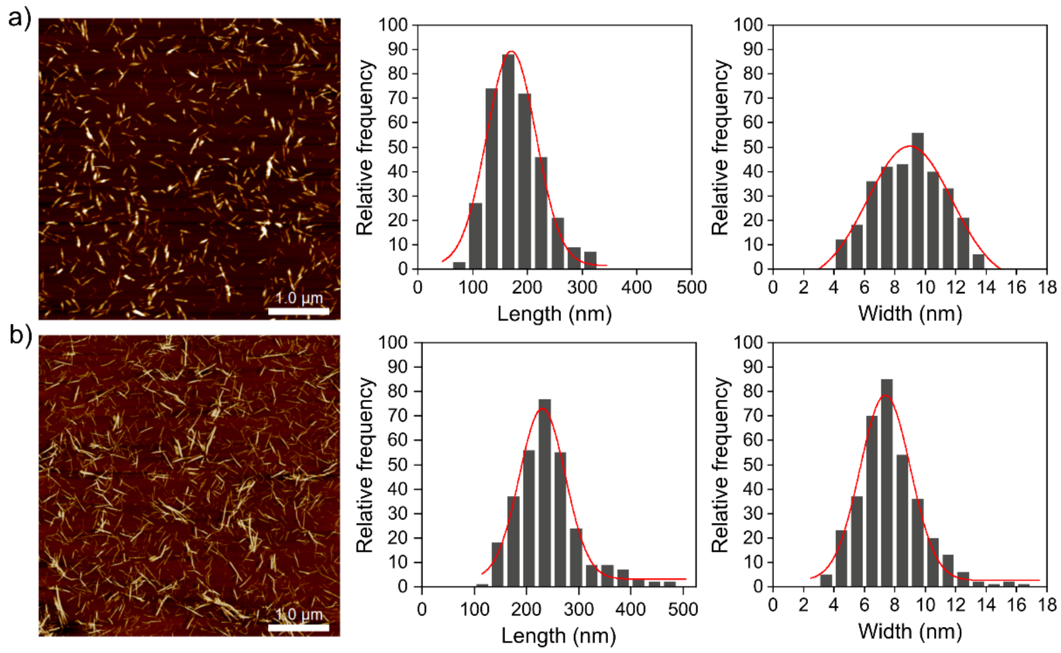

Figure S20. AFM height images for (a) the wood CNCs and (b) partially deacetylated ChNCs, and corresponding histograms showing their width and length distributions.

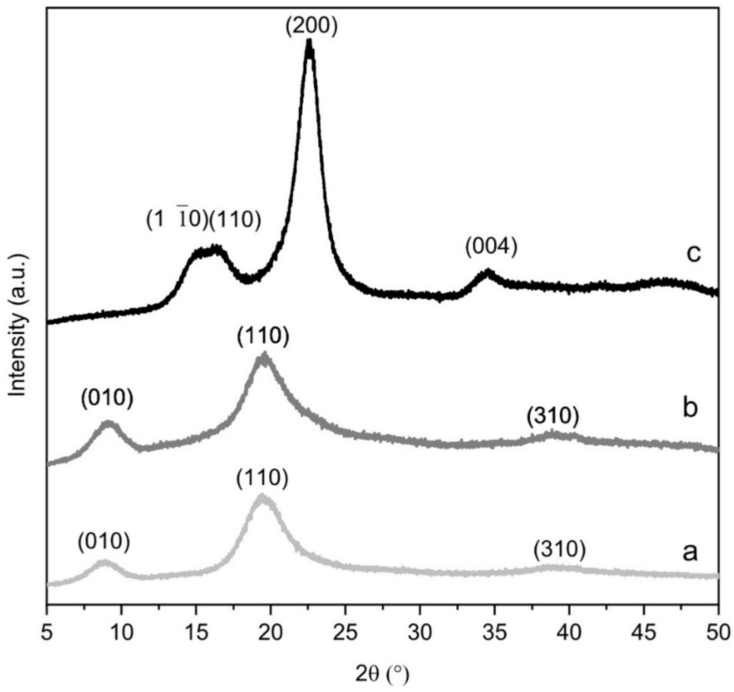

Figure S21. XRD pattern of (a) the extracted squid chitin, (b) partially deacetylated ChNCs, and (c) the wood CNCs.

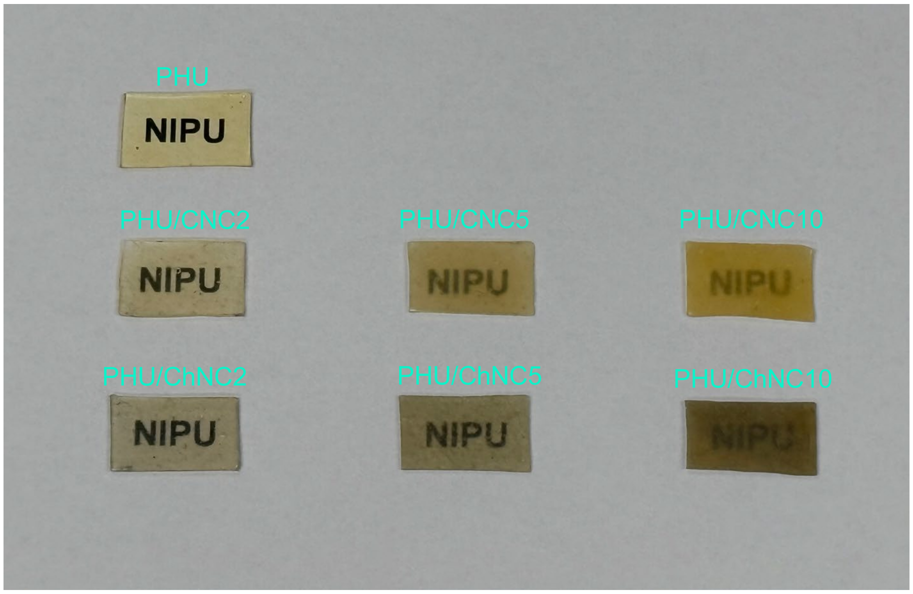

Figure S22. Photographs of the neat PHU, PHU/CNC and PHU/ChNC nanocomposite films on the printed letters of NIPU.

3. Thermal and mechanical properties of the composites.

Table S1. The temperatures at 5% mass lost ($T_{5\%}$), the maximum mass loss temperatures (T_{max1} and T_{max2}), and the char residues at 600 ºC for the neat PHU, PHU/CNC and PHU/ChNC nanocomposites from TGA analysis.

Sample	Filler content (wt%)	$T_{5\%}$ (°C)	T_{max1} (°C)	T_{max2} (°C)	Char Residue at 600 °C
PHU	0	290	324	417	2.5
PHU/CNC2	2	295	346	418	3.0
PHU/CNC5	5	297	353	418	4.4
PHU/CNC10	10	292	350	419	6.3
PHU/ChNC2	2	295	352	417	5.6
PHU/ChNC5	5	286	354	417	6.3
PHU/ChNC10	10	283	350	418	8.8

Table S2. Young's modulus, ultimate tensile strength, and strain at break of the neat PHU, the PHU/CNC, and PHU/ChNC nanocomposites from tensile test.

Sample	Young's modulus (MPa)	Ultimate tensile strength (MPa)	Strain at break (%)
PHU	0.4 ± 0.1	0.14 ± 0.08	1320 ± 356
PHU/CNC2	1.0 ± 0.2	0.13 ± 0.03	1597 ± 342
PHU/CNC5	1.3 ± 0.2	0.18 ± 0.01	745 ± 130
PHU/CNC10	1.2 ± 0.3	0.13 ± 0.01	367 ± 129
PHU/ChNC2	2.9 ± 0.3	0.39 ± 0.05	393 ± 62
PHU/ChNC5	11.0 ± 0.6	0.90 ± 0.16	92 ± 32
PHU/ChNC10	58.8 ± 3.4	2.80 ± 0.40	43 ± 8
PHU/ChNC-S10	25.6 ± 2.5	1.20 ± 0.11	14 ± 4

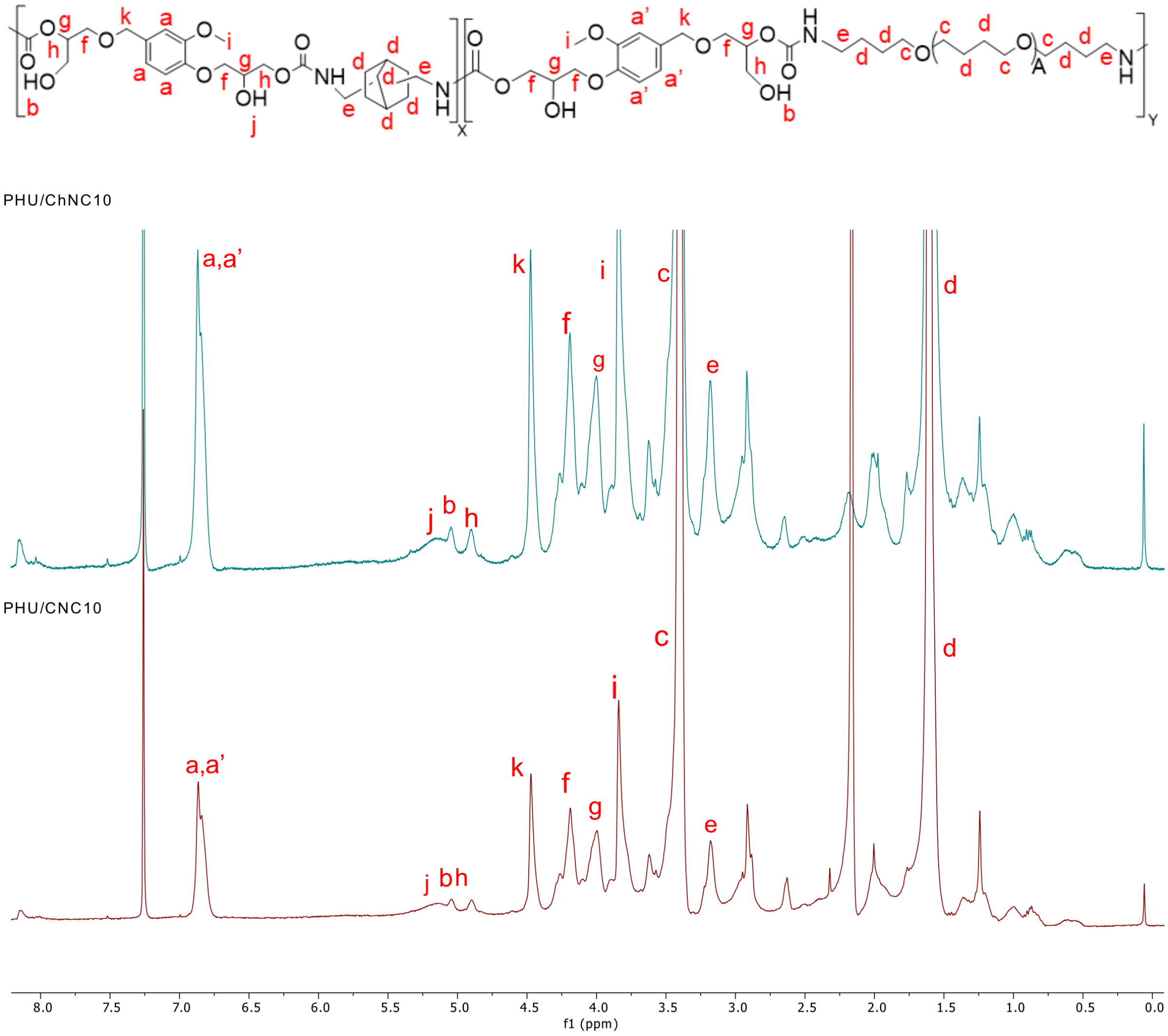

Figure S23. ^{1}H NMR Spectra of PHU/ChNC10 and PHU/CNC10 nanocomposite samples in $CDCl_3$.

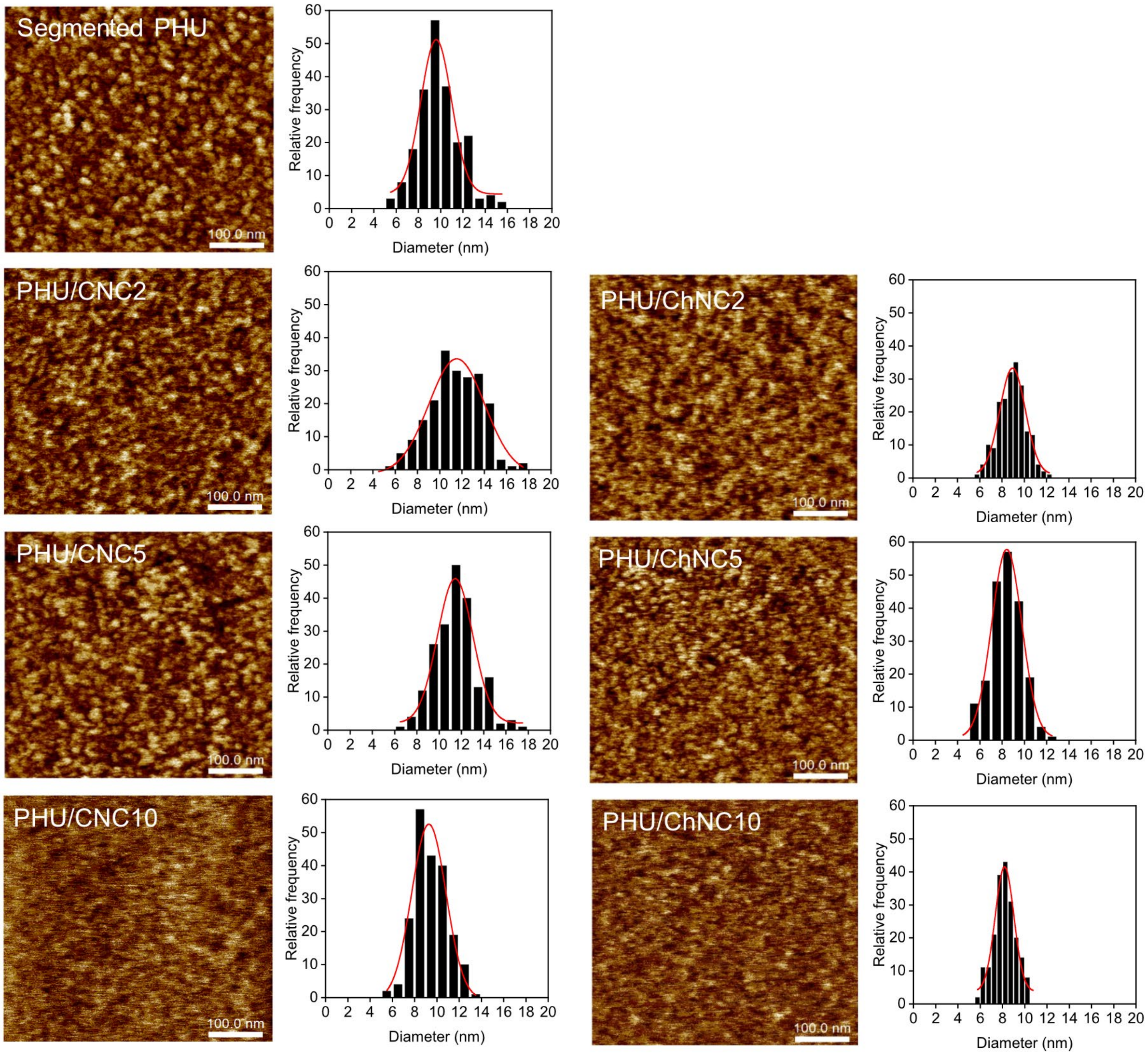

Figure S24. AFM inphase images of the cross-sectional surfaces of the neat segmented PHU and the PHU/CNC and PHU/ChNC nanocomposites and corresponding histograms showing hard domain sizes (average diameters).
